# Structural basis of colibactin activation by the ClbP peptidase

**DOI:** 10.1038/s41589-022-01142-z

**Published:** 2022-10-17

**Authors:** José A. Velilla, Matthew R. Volpe, Grace E. Kenney, Richard M. Walsh, Emily P. Balskus, Rachelle Gaudet

**Affiliations:** 1 Department of Molecular and Cellular Biology, Harvard University, Cambridge, MA USA; 2 Department of Chemistry and Chemical Biology, Harvard University, Cambridge, MA USA; 3 Harvard Cryo-EM Center for Structural Biology, Harvard Medical School, Boston, MA USA; 4 Department of Biological Chemistry and Molecular Pharmacology, Blavatnik Institute, Harvard Medical School, Boston, MA USA; 5 Howard Hughes Medical Institute, Harvard University, Cambridge, MA USA

**Keywords:** Proteases, Enzyme mechanisms, Natural products, X-ray crystallography

## Abstract

Colibactin, a DNA cross-linking agent produced by gut bacteria, is implicated in colorectal cancer. Its biosynthesis uses a prodrug resistance mechanism: a non-toxic precursor assembled in the cytoplasm is activated after export to the periplasm. This activation is mediated by ClbP, an inner-membrane peptidase with an N-terminal periplasmic catalytic domain and a C-terminal three-helix transmembrane domain. Although the transmembrane domain is required for colibactin activation, its role in catalysis is unclear. Our structure of full-length ClbP bound to a product analog reveals an interdomain interface important for substrate binding and enzyme stability and interactions that explain the selectivity of ClbP for the *N*-acyl-d-asparagine prodrug motif. Based on structural and biochemical evidence, we propose that ClbP dimerizes to form an extended substrate-binding site that can accommodate a pseudodimeric precolibactin with its two terminal prodrug motifs in the two ClbP active sites, thus enabling the coordinated activation of both electrophilic warheads.

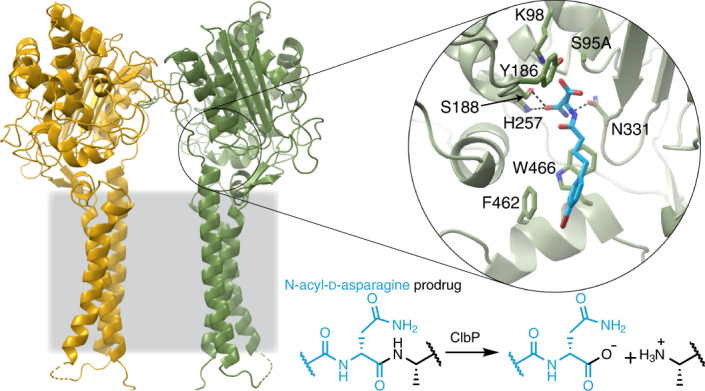

## Abstract (web summary)

Structure and mutagenesis of the colibactin-activating peptidase ClbP reveals a dimer with a substrate-binding transmembrane domain and a conserved polar network in its periplasmic domain that enforces selectivity for d-asparagine prodrug motifs.

## Main

Colibactin is a small-molecule genotoxin produced by gut bacteria and extra-intestinal pathogenic strains, and it is implicated in diseases such as inflammatory bowel disease and colorectal cancer^1,2^. Inflammation in the host is proposed to potentiate the genotoxicity of colibactin in the gut, resulting in DNA inter-strand cross-linking and genomic instability^3–5^. Colibactin is biosynthesized by a hybrid non-ribosomal peptide synthetase–polyketide synthase (NRPS–PKS) assembly line encoded by the *pks* genomic island (the *clb* gene cluster)^1,6,7^. A highly reactive natural product, colibactin has never been isolated from the producing bacteria. Current knowledge about its chemical structure combines insights from genetics, biochemistry, total synthesis and DNA adductomics^8^.

**Fig. 1:**
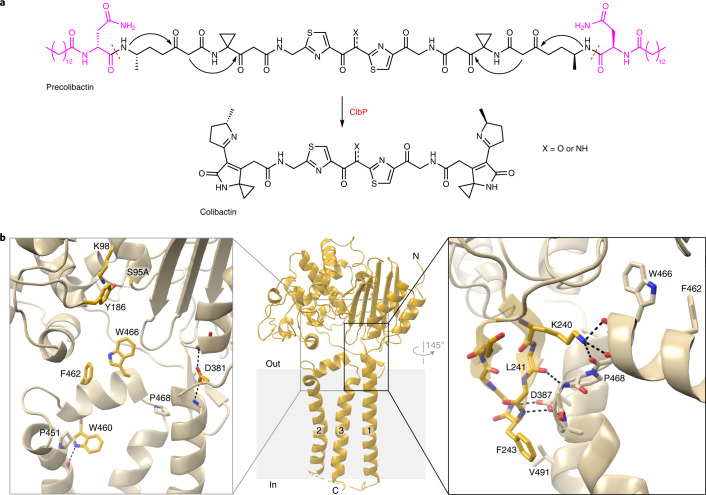
The TMD of ClbP completes the substrate-binding site. **a**, The proposed structure of colibactin is pseudodimeric and contains two electrophilic warheads that generate inter-strand cross-links in the DNA of epithelial cells in the human gut. To activate this toxin, the ClbP peptidase cleaves off the two prodrug motifs (colored in magenta) from the precursor molecule precolibactin, leading to non-enzymatic condensation to form the active warheads (curved arrows). **b**, The structure of full-length ClbP reveals an interface between the periplasmic and transmembrane domains. The inset on the left provides an expanded view of the interdomain interface. The conserved TMD residues and the catalytic triad are shown as sticks. The inset on the right shows interactions of the β3-β4 loop (dark yellow) with the TMD that likely stabilize the orientation of the catalytic site toward the cell membrane.

Colibactin is proposed to be a pseudodimeric molecule containing two DNA-alkylating warheads connected by a central linker that likely degrades rapidly in the presence of molecular oxygen (Fig. 1a)^9,10^. Each warhead consists of an electrophilic α,β-unsaturated imine-conjugated cyclopropane that reacts primarily with adenines at the N3 position^11,12^. Reaction of the two warheads with DNA creates inter-strand cross-links, leading to double-strand breaks and ultimately to mutational signatures that have been detected in human cancer genomes^13,14^.

Colibactin-producing bacteria control warhead formation through a prodrug resistance mechanism to protect their own DNA^15^. In this biosynthetic strategy, cytoplasmic enzymes assemble a non-toxic precursor (precolibactin) containing two *N*-myristoyl-d-asparagine prodrug motifs. Precolibactin is then exported to the periplasm where it is converted to the active genotoxin by the ClbP peptidase. Cleavage of each prodrug motif by ClbP exposes a primary amine that undergoes a non-enzymatic condensation to yield the imine essential for colibactin-induced DNA damage (Fig. 1a)^11,16^. Other toxic bacterial natural products, including amicoumacin, xenocoumacin and zwittermicin, share the *N*-acyl-d-asparagine prodrug motif biosynthetic strategy and are activated by homologous membrane-bound peptidases^17^, although the active toxins are otherwise structurally unrelated to colibactin.

ClbP is an inner-membrane d-amino peptidase that contains an N-terminal periplasmic catalytic domain followed by a three-helix transmembrane domain (TMD). ClbP and its prodrug-activating peptidase homologs belong to the S12 family of serine hydrolases defined by conserved SxxK and YxN catalytic motifs and including class C β-lactamases, such as AmpC (refs. ^18,19^). ClbP residues S95, K98 and Y186 are indispensable for enzymatic activity, and, in a crystal structure of the periplasmic domain, these residues converge to form the catalytic site^19^. ClbP cleaves precolibactin analogs with varied acyl chains and amide substituents but is highly specific toward the d-asparagine sidechain^20^. In the absence of substrate-bound structures, the mechanism underlying the substrate specificity of ClbP is not yet known. Compared to other S12 homologs, the ClbP periplasmic domain has distinguishing features, such as a broad substrate-binding site and a predominantly negative electrostatic surface^19,21^. However, the isolated periplasmic domain is catalytically inactive, and, although only one TM helix is necessary for insertion into the inner membrane, all three are required for prodrug cleavage in colibactin biosynthesis^21^.

To investigate the role of the TMD in ClbP function and elucidate the structural basis of substrate specificity, we determined a crystal structure of full-length catalytically inactive S95A bound to a product analog. From this structure, we infer that the prodrug motif binds at the interface between the periplasmic and transmembrane domains, and TMD residues, especially W466, are crucial for peptidase activity in vitro. The d-asparagine sidechain in the prodrug motif hydrogen-bonds with S188 and N331, which are critical for peptidase activity in vitro. Changes to the hydrogen-bonding network which orients the N331 sidechain alter substrate specificity, demonstrating its importance for d-asparagine recognition. Crystal-packing interactions suggest that ClbP forms a dimer, and we confirmed this same dimeric species in solution using single-particle cryogenic electron microscopy (cryo-EM). In the dimer, the two periplasmic domains form a canopy over the cell membrane under which the active sites face each other. We docked the proposed precolibactin structure onto the binding surface subtended by this canopy to illustrate that precolibactin can bind to ClbP with the two terminal prodrug motifs occupying the active sites of each subunit simultaneously. ClbP dimerization further supports the proposed pseudodimeric precolibactin structure and suggests a potential regulatory role in colibactin production and bioactivity: simultaneous activation of the two warheads may increase the probability that the toxin produced has two reactive sites capable of cross-linking DNA.

## Results

### The ClbP TMD extends the substrate-binding cleft

To better understand the role of the ClbP TMD in enzymatic function, we determined the crystal structure of full-length ClbP embedded in a lipid mesophase. Seeking structures in the presence of substrate analogs, we initially focused on the catalytically inactive S95A variant^19^. Early S95A crystals yielded electron density maps that clearly showed the position of individual transmembrane helices, although their resolution was insufficient to assign the registry or helix connectivity pattern. To overcome this challenge, we mutated non-conserved TMD positions to methionine—L454M and I478M—and used anomalous diffraction data from selenomethionine-substituted crystals to locate these sequence markers in electron density maps. We obtained two high-resolution structures of S95A-L454M-I478M: selenomethionine-substituted ClbP and methionine-containing ClbP bound to a product analog (Supplementary Table 1). The resulting high-quality maps allowed us to fully model the TMD helices and unambiguously assign their registry (Supplementary Fig. 1a,b). The two structures are nearly identical (root mean squared deviation (RMSD) = 0.15 Å over the Cα carbons of residues 38–414 and 431–491), except for the extent to which the TMD’s intracellular loops are modeled; we base our analysis below on the higher-resolution product-bound structure. Our structure of wild-type ClbP presented in an accompanying paper^22^ has an overall RMSD of 0.35 Å and 0.37 Å in the TMD (residues 382–409 and 431–491) to the unbound and product-bound structures, respectively, confirming that the introduced mutations do not affect the structure (Supplementary Fig. 1c).

In full-length ClbP, the interaction between the periplasmic and transmembrane domains extends the substrate-binding surface and orients the catalytic residues toward the cell membrane. The periplasmic domain sits atop the TMD, with most of its structure virtually identical to the previous isolated domain structure (RMSD 0.33 Å over 288 Cα atoms; Supplementary Fig. 1d). The TMD connects to the periplasmic domain through an 11-residue loop and consists of a three-helix bundle with TM3 in the center interacting with both TM1 and TM2 and no contacts between TM1 and TM2 (Fig. 1b). The periplasmic TM2–TM3 linker includes a two-turn helix that sits underneath the catalytic residues, whereas the cytoplasmic TM1–TM2 linker is disordered and unresolved in our structures. F462 and W466 in the TM2–TM3 linker protrude toward the catalytic residues (Fig. 1b), forming an extended substrate-binding cleft as detailed below. The β3-β4 loop in the periplasmic domain (residues 218–254) appears to dip into the membrane to interact with the TMD and is the only region that is different in the isolated periplasmic domain structure (Supplementary Fig. 1d). The interactions of the β3-β4 loop with the TMD likely help orient the catalytic site toward the cell membrane, where precolibactin is presumably anchored by its two myristoyl chains. Specifically, K240 interacts with and satisfies the C-terminal negative dipole of the TM2–TM3 linker helix, an interaction that may be particularly important in the hydrophobic membrane environment. F243 also contributes to this clasp, forming non-polar contacts with residues in TM1 (Fig. 1b).

**Extended Data Fig. 1:**
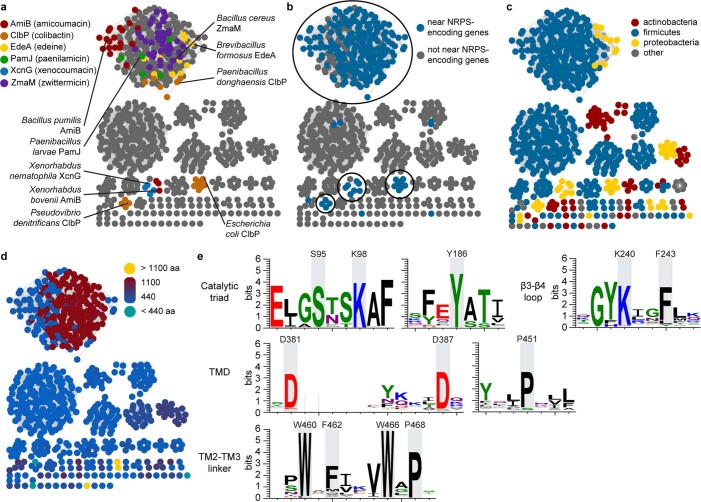
Identification and sequence conservation of prodrug-activating homologs of ClbP. **a**, Sequence similarity network (SSN) for 730 ClbP homologs, colored by identified BGC (if any). Peptidases involved in amicoumacin, edeine, paenilamicin, and zwittermicin biosynthesis cluster together, along with the newly identified probable Gram-positive colibactin producers. The Gammaproteobacterial ClbPs are split into two distinct subsets, one comprising close relatives of the sequences found in *Pseudovibrio* strains^56–59^ and the other representing ClbPs from BGCs with canonical architecture in *Escherichia* species (with homologs from *Erwinia*^60^, *Frischella*, *Gilliamella*, and *Serratia* strains^56^, among others). Similarly, AmiB homologs in *Xenorhabdus* strains cluster with XcnG sequences rather than Gram-positive AmiBs. Intriguingly, the genomes of some strains – such as the edeine-producing *B. formosus* NF2 – appear to have multiple biosynthetic gene clusters containing authentic prodrug peptidases with potentially distinct activities. **b**, SSN colored to highlight proximity (within a ±10 gene neighborhood) of the peptidase gene to a gene containing both NRPS A and C domains, as a proxy for the presence of a NRPS module playing a ClbN-like role. The only SSN clusters in which this condition was met were clusters containing homologs of known prodrug peptidases (circled in black). All our sequence conservation analyses were performed using these clusters. **c**, SSN colored by phylum, highlighting that prodrug-activating peptidases are most common among Firmicutes, with some spread into Proteobacteria (as seen with the *ami*, *clb*, and *xcn* BGCs) and into Actinobacteria. **d**, The prodrug-activating peptidase SSN is colored by amino acid sequence length to emphasize that a large subset of sequences (including EdeA, PamJ, and ZmaM homologs) are much longer. This can be attributed to fusion with a second domain with homology to components of an ABC transporter, commonly annotated as a cyclic peptide transporter. However, Gram-positive AmiB and ClbP sequences (and other unidentified but related proteins) lack this additional domain and more closely resemble *E. coli* ClbP, *X. bovenii* AmiB, and XcnG. **e**, Sequence logos built from the alignment of 271 candidate prodrug-activating peptidases detail sequence conservation of the catalytic triad and of periplasmic-TMD interface and intra-TMD positions discussed in the main text.

To investigate the sequence conservation of positions across the interdomain interface among candidate prodrug-activating peptidases, we searched the UniProt database for members of the PF00144 (β-lactamase) superfamily that had at least two transmembrane helices and were 400–1,200 amino acids in length, consistent with similarity to either ClbP or the related prodrug-activating peptidase ZmaM (which contains an ABC half-transporter domain fused C-terminal to the ClbP-like peptidase). We built sequence similarity networks (SSNs) from protein sequences encoded by genes for which a genomic neighborhood of at least ten genes in either direction was available, using representative nodes at 100% identity to eliminate identical sequences and an expectation value cutoff of 1 × 10^−90^. Based on the genomic neighborhoods, we identified four clusters within the SSN containing ClbP homologs from amicoumacin^23^, colibactin^1,6,7^, edeine^24^, paenilamicin^25^, xenocoumacin^26^ and zwittermicin^27^ biosynthetic gene clusters (Supplementary Fig. 2 and Extended Data Fig. 1a,b). These homologs are mostly in Firmicutes but also in Proteobacteria (Extended Data Fig. 1c), and their length is either ClbP-like (~450 amino acids) or ZmaM-like (~1,100 amino acids) (Extended Data Fig. 1d). These analyses also suggest that several Gram-positive *Paenibacillus* strains produce a compound highly similar to colibactin and that edeine biosynthesis employs an *N*-acyl-d-asparagine prodrug peptidase mechanism. This hypothesis is validated in an accompanying paper, in which a ClbP inhibitor stimulates accumulation of newly identified preedeines in a *Brevibacillus* strain^22^. Other SSN clusters were not part of gene clusters encoding NRPS machinery (Extended Data Fig. 1b) and are, therefore, unlikely to be involved in prodrug biosynthesis systems. We excluded sequences from these clusters from further analyses.

In our alignment of 271 prodrug-activating peptidases, seven TMD residues are highly conserved (Extended Data Fig. 1e). First, the two TM2–TM3 linker residues facing the catalytic site are conserved—W466 is strictly conserved, whereas neighboring F462 varies more, with substitutions to other hydrophobic residues and serine or threonine. W460 in the TM2–TM3 linker is the only other strictly conserved TMD residue. Its sidechain is wedged between TM2 and TM3 under the TM2–TM3 linker helix, with its indole amine group hydrogen-bonding to the L447 backbone carbonyl, buttressing a kink in TM2 at a conserved proline, P451 (Fig. 1b). Another conserved proline, P468, bridges the linker helix and TM3. The last two conserved TMD residues stabilize the interdomain interface: D381 caps the N-termini of helix α11 and TM1 (Fig. 1b), and D387 in TM1 hydrogen-bonds with two backbone amides of the β3-β4 loop hairpin turn (Fig. 1b). Two periplasmic domain residues noted above as playing a role in the interdomain interface are also highly conserved: K240 in the β3-β4 loop is largely conserved as a lysine, although it is sometimes substituted to other polar residues, and F243 is conserved as a large hydrophobic residue (Extended Data Fig. 1e). Overall, the conservation patterns suggest a stable relative orientation of the two domains and an important role for the extended substrate-binding cleft at the interdomain interface in all prodrug-activating peptidases.

### The prodrug motif binds at the interdomain interface

**Fig. 2:**
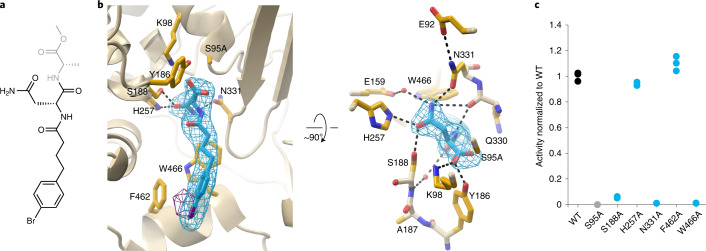
The prodrug motif binds at the interface between periplasmic and transmembrane domains. **a**, Substrate analog included in crystallization of catalytically inactive ClbP. Our data suggest that this molecule is hydrolyzed during crystallization, as the atoms in gray are not observed in the electron density map. **b**, Two views, related by a 90° rotation, of the hydrolysis product bound at the active site. The d-asparagine sidechain of the prodrug motif interacts with periplasmic domain residues S188, H257 and N331, and the acyl chain interacts with TM2–TM3 linker residues F462 and W466 (sidechains shown as sticks). Polder map omitting the product contoured at 7σ is colored in cyan, and bromine anomalous difference Fourier map contoured at 3.5σ is colored in purple. **c**, Enzymatic activity of purified ClbP variants measured as cleavage of a fluorogenic substrate analog (Extended Data Fig. 3c). The plot represents triplicate measurements normalized to the average for wild-type (WT) ClbP. Source data

**Extended Data Fig. 2:**
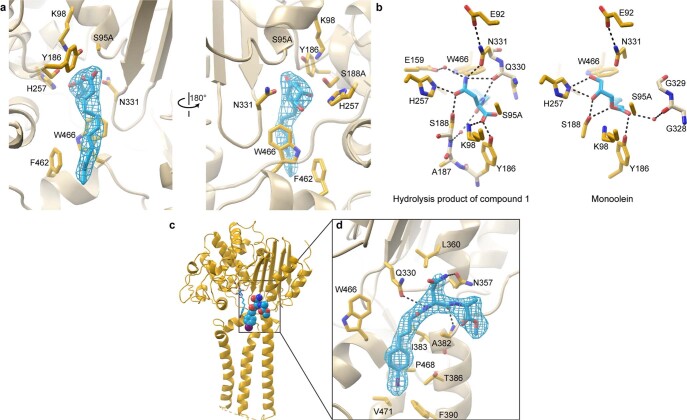
Comparison of the structures of ClbP in the presence of monoolein and a product analog illustrates that monoolein mimics a ClbP substrate or product. **a**, Monoolein in the crystallization mesophase was trapped in the active site of one of our structures. The panels show two views, related by a 180° rotation, of a monoolein molecule (cyan) bound in the active site, with the corresponding electron density for a polder map contoured at 7σ. **b**, A side-by-side comparison of the active-site interactions of the (4-(4-bromophenyl)butanoyl)-d-asparagine product and monoolein illustrates that monoolein interacts similarly with active site residues that bind to the prodrug motif, explaining how it can outcompete substrate analogs introduced only in the precipitant solution but not the lipidic mesophase during the crystallization process. Hydrogen bonds are indicated as black dotted lines. **c**, In addition to the hydrolysis product in the active site (cyan sticks), we observed electron density corresponding to an intact substrate molecule at an adjacent site (cyan spheres). **d**, The inset shows sidechains within 4.2 Å of the bound substrate analog, with hydrogen bonds indicated as black dotted lines. The corresponding electron density for a polder map is contoured at 7σ.

We determined the structure of catalytically inactive ClbP with a bound substrate analog containing the *N*-acyl-d-asparagine prodrug motif. Because molecules containing the myristoyl substituent found in precolibactin are poorly soluble, we used an analog that replaces the myristoyl chain with a 4-phenylbutyryl group (compound **1**, *N*-4-(4-bromophenyl)butanoyl-d-asparaginyl-l-alanine methyl ester; Fig. 2a), which is more soluble and is processed as effectively as myristoylated analogs^20^. We introduced a bromine substituent at the *para* position of the phenyl ring as an anomalous scatterer to validate the presence of the substrate analog in the electron density maps and aid in model building. We initially crystallized S95A-L454M-I478M in a precipitant solution supplemented with compound **1** and obtained a 2.7-Å structure that suggested that monoolein, the crystallization lipid, was bound at the active site (Extended Data Fig. 2a). In our model, the headgroup of monoolein forms polar interactions with active site residues S188 and H257. The chemical similarity to myristoyl-d-asparagine suggests that monoolein acts as a substrate mimic (Extended Data Fig. 2b). After supplementing compound **1** in both the protein–lipid bolus and the precipitant, we obtained high-resolution electron density maps consistent with the presence of the hydrolysis product of the analog in the active site and an intact substrate molecule in an adjacent site (Fig. 2b and Extended Data Fig. 2c,d). We validated the presence of this hydrolysis product in the active site using anomalous difference Fourier maps calculated from diffraction data collected at the bromine absorption edge (Fig. 2b).

The carboxylate of the hydrolysis product, which corresponds to the scissile bond of the substrate, is next to A95 with an oxygen atom hydrogen-bonding with catalytic triad residues K98 and Y186 (2.8 Å and 2.4 Å respectively; Extended Data Fig. 2b). The N-terminal hydrophobic group of the product contacts TMD residues F462 and W466, further supporting their role in binding the acyl chain of the prodrug motif (Fig. 2b). The longer native myristoyl group could extend along the transmembrane helices of ClbP and reach the membrane lipids, suggesting that precolibactin can reach the ClbP active site by diffusion while embedded in the inner membrane’s outer leaflet. The d-asparagine sidechain crucial for substrate recognition by ClbP interacts with periplasmic domain residues S188, H257 and N331. The d-asparagine sidechain carbonyl hydrogen-bonds with S188 and H257, whereas its sidechain amino group hydrogen-bonds with the N331 sidechain carbonyl (Fig. 2b). The orientation of the N331 sidechain amide is itself set by a hydrogen bond between its amino group and E92, which is, in turn, stabilized by a salt bridge with K235.

**Extended Data Fig. 3:**
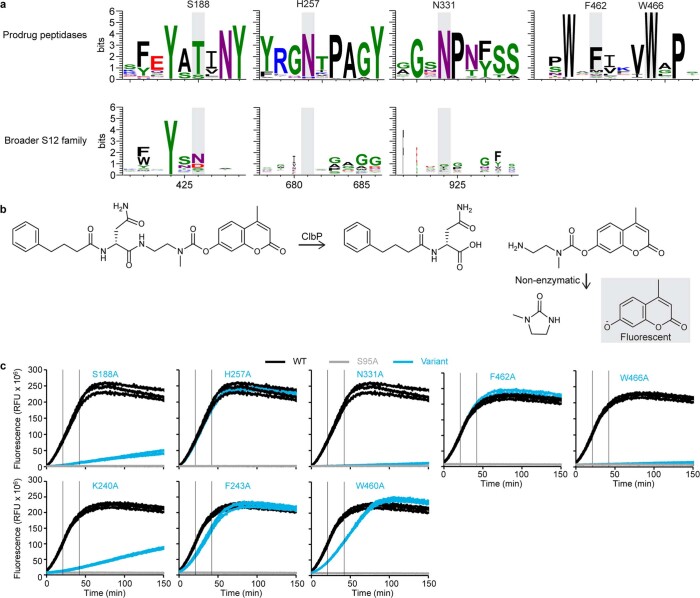
Activity of ClbP variants with mutations to conserved substrate-binding residues measured using a fluorogenic assay. **a**, Sequence logos representing conservation of *N*-acyl-d-asparagine binding residues among 271 aligned sequences of prodrug-activating homologs (top), compared to logos built from an alignment of 901 representative sequences from the broader S12 family downloaded from the MEROPS database. **b**, Fluorogenic activity assay used to measure the peptidase activity of ClbP variants^20^. Cleavage of the synthetic substrate probe by ClbP generates an intermediate which then undergoes a non-enzymatic cyclization reaction to yield the active fluorophore (gray box). **c**, Curves of the raw fluorescence versus time for different ClbP variants with point mutations at residues of interest that interact with the substrate (top row) or form notable interdomain (K240A and F243A) or intra-TMD (W460) interactions (see Extended Data Fig. 1e for the corresponding sequence logos). Each panel represents triplicate measurements for the indicated variant (cyan). For comparison, the corresponding triplicate measurements for wild-type ClbP (black) and catalytically inactive S95A (gray), measured in the same experiment, are reproduced on each graph. The two gray vertical lines bound the data used for calculating the normalized hydrolysis rates in Fig. 2. Source data

Although N331 is strictly conserved among putative prodrug-activating peptidases, it is not conserved among the wider S12 family, suggesting that this position is important for d-asparagine binding in the xenocoumacin, amicoumacin, zwittermicin, paenilamicin and edeine peptidases (Extended Data Fig. 3a). Similarly, position 188 is conserved as a serine or threonine in prodrug-activating peptidases, and position 257 is a histidine or an asparagine. In contrast, position 188 is highly conserved as an asparagine among the broader S12 family, whereas H257 is not conserved. The high conservation of the d-asparagine-binding residues specifically among prodrug-activating peptidases supports an important role for these residues in substrate specificity.

### N331 and W466 are essential for prodrug cleavage

We explored the role of interactions between the prodrug motif and its binding site on ClbP’s enzymatic function by measuring cleavage of a fluorogenic substrate analog by purified ClbP variants (Extended Data Fig. 3b,c)^20^. TM2–TM3 linker mutation F462A did not affect enzyme activity, whereas the W466A mutation completely abrogated activity, demonstrating that W466 is essential for peptidase activity in vitro (Fig. 2c).

Mutating d-asparagine-binding residue N331 to alanine also abolished ClbP activity. Similarly, mutating S188 to alanine severely impaired activity. Thus, the hydrogen bonds of these two highly conserved residues to d-asparagine are critical for robust peptidase activity (Fig. 2c). The activity of H257A was similar to wild-type, suggesting that the interaction mediated by this residue is not required for peptidase activity (Fig. 2c).

Finally, we examined whether residues at the interdomain interface but not directly involved in substrate binding have a role in enzyme activity. Substitutions F243A in the β3-β4 loop and W460A in the TM2-TM3 linker had no effect, whereas K240A severely reduced activity (Extended Data Fig. 3c). Its consistently lower expression levels and purification yields suggest that K240A impairs activity by disrupting protein stability rather than directly affecting substrate binding or catalysis. These results support an important role for the interactions of K240 and the TM2–TM3 helix—and by extension, interdomain interactions—in ClbP protein stability.

### N331 enforces d-asparagine specificity

**Fig. 3:**
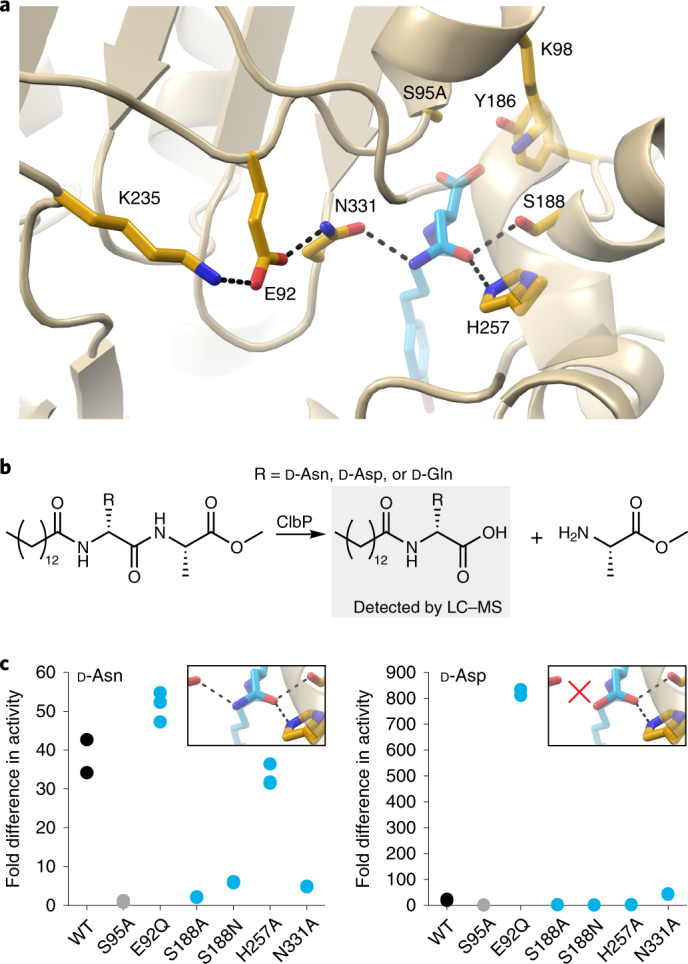
N331 enforces d-asparagine specificity. **a**, A network of interactions initiated by K235 orients N331 such that the carbonyl in its sidechain faces toward the binding pocket (the cartoon representation of residues 255–261 is transparent to optimize the view). **b**, Activity assay with substrate analogs containing prodrug motifs with alternative d-amino acids. Cleaved prodrug motif is detected by LC–MS (normalized to AUC of S95A) after a 5-hour incubation of the substrate with purified ClbP variants. **c**, Results of the assay in **b** for the substrate analogs containing d-Asn (left) or d-Asp (right); *n* = 3 independent experiments. None of the ClbP variants cleaved substantial amounts of the d-Gln-containing substrate analog (Extended Data Fig. 4d). Perturbing the orientation of the N331 sidechain allows ClbP to cleave d-aspartate substrates, suggesting that this residue is crucial for substrate specificity. Representative traces from the LC–MS are shown in Extended Data Fig. 4c. WT, wild-type. Source data

**Extended Data Fig. 4:**
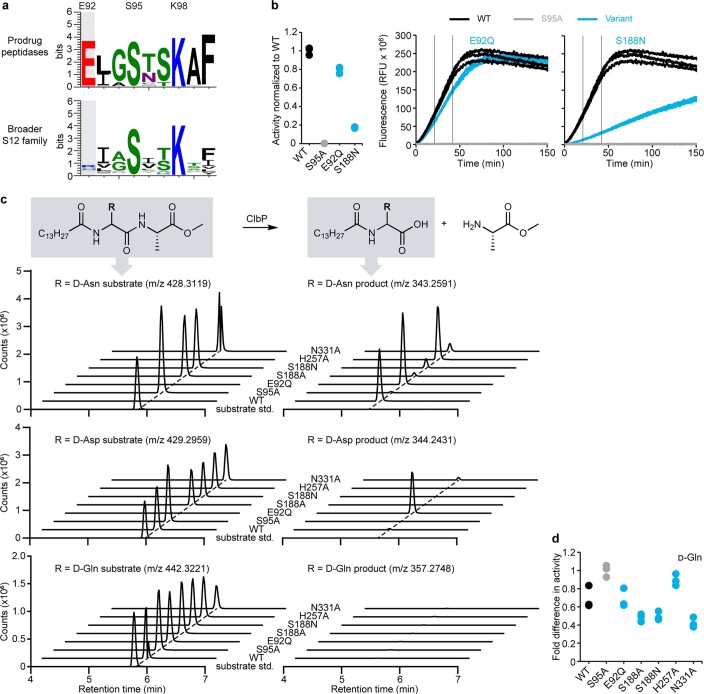
Wild-type ClbP and variants with mutations at d-asparagine binding residues cannot process substrate with an *N*-acyl-d-glutamine prodrug motif. **a**, Sequence logo representing conservation of E92, which stabilizes the orientation of d-asparagine specificity residue N331. **b**, Normalized hydrolysis rates calculated from activity assays performed with d-asparagine binding mutants E92Q and S188N (left). Triplicate fluorescence activity measurements for each mutant (cyan) are shown with triplicates for wild-type ClbP (black) and catalytically inactive S95A (gray) collected in the same experiment (right). **c**, Assay measuring activity of mutants for dipeptide substrates containing d-asparagine, d-aspartate, or d-glutamine prodrug motifs by LC–MS detection of cleaved product. Extracted Ion Chromatogram (EIC) traces of the [M + H]^+^ ion for each of the substrates tested (left) and the expected ClbP cleavage product (right). **d**, None of the d-asparagine binding mutants process substantial amounts of the d-glutamine substrate, as indicated by the lack of a difference in activity between the catalytically deficient S95A and any of the other variants (n = 3 independent experiments). Source data

We hypothesized that N331 enforces the specificity of ClbP for substrates with d-asparagine prodrug motifs and that its sidechain orientation, set by a hydrogen bond with E92 to present a hydrogen bond-accepting carbonyl to the substrate, is responsible for excluding d-aspartate analogs, which are cleaved at marginal rates by ClbP (ref. ^20^). To test this hypothesis, we perturbed the interaction network determining the N331 orientation (Fig. 3a) by mutating E92 to glutamine. We compared the hydrolase activity of wild-type and E92Q toward substrates with d-asparagine and d-aspartate prodrug motifs using a liquid chromatography–mass spectrometry (LC–MS) assay (Fig. 3b)^20^. This mutation indeed broadened substrate specificity: E92Q had wild-type-like activity for the d-asparagine substrate, and it also processed the d-aspartate substrate effectively, whereas wild-type ClbP did not (Fig. 3c). In contrast, S188A, H257A and N331A did not cleave substantial amounts of the d-aspartate substrate, and their behavior toward the d-asparagine substrate recapitulated the results from our fluorogenic assay (Fig. 3c). This suggests that E92, highly conserved among prodrug-activating peptidases but not across the S12 family (Extended Data Fig. 4a), orients hydrogen-bonding groups in the substrate-binding pocket of ClbP and other prodrug-activating peptidases to provide selectivity toward d-asparagine-containing substrates.

Because mutations at position 188 were implicated in changes to substrate specificity in AmpC (ref. ^28^), we explored the effect of restoring the S12 family consensus asparagine on the specificity of ClbP. S188N still cleaved the d-asparagine substrate but did not process d-aspartate and d-glutamine substrates (Fig. 3c and Extended Data Fig. 4b), indicating that this mutation does not broaden the specificity of ClbP. Activity of S188N for the d-asparagine substrate was lower than wild-type but higher than S188A, suggesting that an asparagine at this position can be a hydrogen-bond donor to the substrate, although the geometry enforced in the bound substrate may not be optimal for catalysis.

### ClbP forms a dimer

**Fig. 4:**
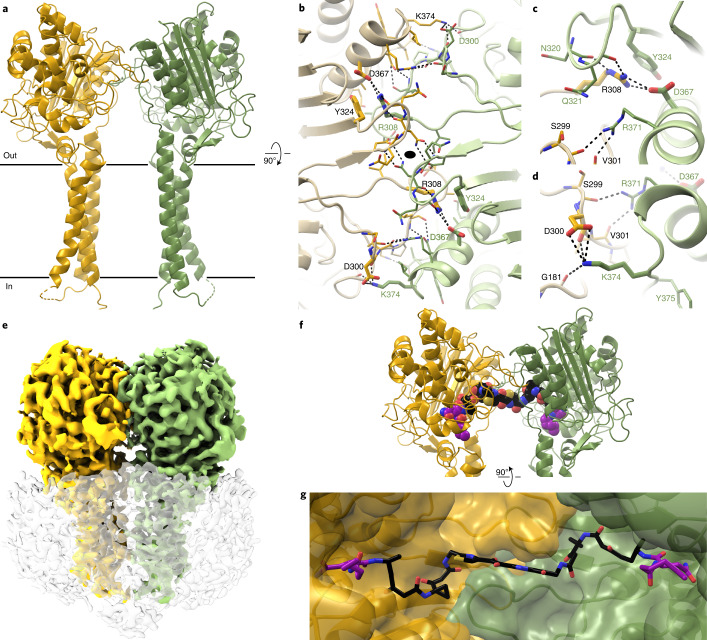
ClbP forms a dimer that accommodates pseudodimeric precolibactin. **a**, ClbP dimer observed in the crystal-packing interactions from a plane perpendicular to the cell membrane, denoted as black lines. **b**, Orthogonal view of the dimer interface looking from the periplasm to the inside of the cell. The interface forms around a two-fold crystallographic symmetry axis (black oval) and consists of a pair of interlocking loops that contribute both hydrophobic and polar interactions. The largest predicted energetic contributors to stabilizing this interface are interactions formed among residues R308, Y324 and D367 (shown as thick sticks). All other residues participating in the interface are shown as thin sticks. **c**,**d**, Detailed view of interactions mediated by R308 (**c**) and K374 (**d**). **e**, 3D reconstruction obtained from cryo-EM analysis of wild-type ClbP. Density colored to correspond to each subunit, and the detergent micelle is shown as a transparent surface with dust hidden for clarity. **f**,**g**, Model of precolibactin binding to the ClbP dimer obtained by individually docking fragments of the molecule (Supplementary Fig. 5). Precolibactin can straddle both subunits of the ClbP dimer such that the prodrug motifs at both ends can each bind a different active site simultaneously. Views of precolibactin binding to the dimer as seen from a plane perpendicular to the membrane (**f**) as well as to the surface of the cavity subtended by the dimer (**g**). Note that the docked molecule contains hexanoyl chains in place of the natural tetradecanoyl (or ‘C_14_’) chains of the myristoyl groups.

**Extended Data Fig. 5:**
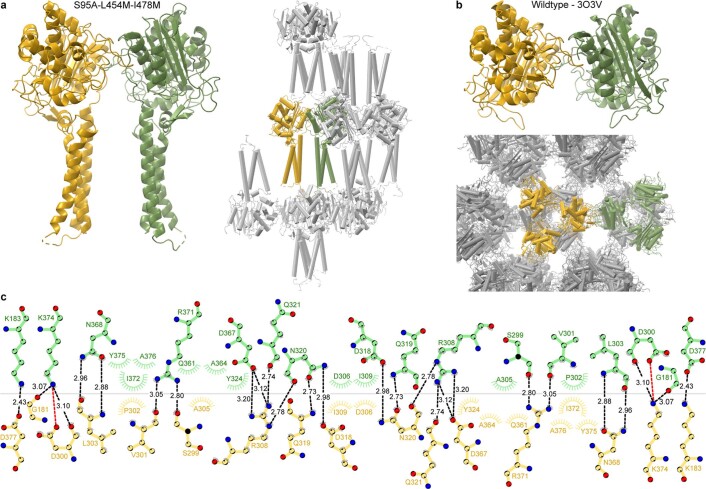
Crystal packing interactions suggest ClbP forms a dimer. **a**, The ClbP dimer (left) and the dimer in context of the crystal packing arrangement (right), with the asymmetric units containing each subunit in yellow or green and other symmetry mates in gray. **b**, The same dimeric arrangement observed in our full-length ClbP structure is also present as a crystallographic dimer interface in the published structure of the isolated periplasmic domain (top), as seen in the interaction between two trimeric asymmetric units (bottom). **c**, 2D representation of the ClbP dimer interface detailing the molecular interactions stabilizing the assembly as well as distances (in Å) between interacting polar groups.

The crystal-packing interactions in our structure include a two-fold symmetric dimer interface that is also present in the structure of the isolated periplasmic domain^19^, which had not been previously described (Fig. 4a). Because the two structures result from different crystal-packing arrangements (Extended Data Fig. 5a,b), we hypothesized that this dimer interface is physiological and that ClbP dimerizes in cells. This hypothesis is particularly relevant in the context of the recently proposed pseudodimeric structure of precolibactin^9,10^. The two ClbP subunits form a dome-shaped canopy over the cell membrane that subtends a largely electronegative surface, which is flanked by the two active sites 35.5 Å apart (distance between S95 γO atoms). The dimer interface in our crystal lattice is centered around a crystallographic two-fold axis and exclusively consists of interactions between periplasmic domains, with two pairs of interlocking loops contributing both polar and hydrophobic contacts (Fig. 4b and Extended Data Fig. 5c). The interface buries 1,285 Å^2^ of surface area per subunit. Several buried polar interactions are predicted as the largest energetic contributors stabilizing this assembly^29^, including those mediated by R308, namely a salt bridge with D367 and a cation–π interaction with Y324 and a salt bridge between K374 and D300 (Fig. 4c,d).

**Extended Data Fig. 6:**
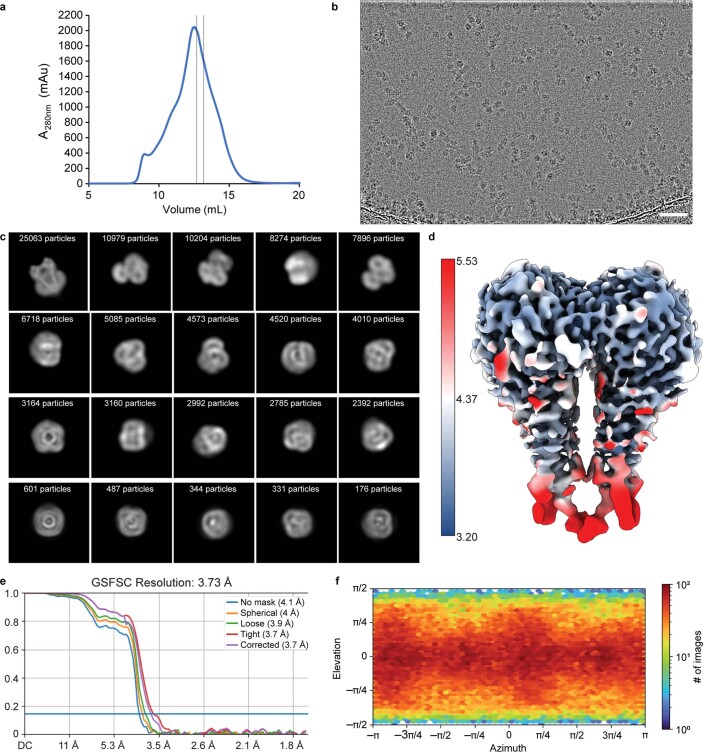
Cryo-EM data analysis of ClbP. **a**, Size exclusion chromatogram of wild-type ClbP purified in GDN. Protein eluted in the fraction bound by the vertical lines was used for cryo-EM analysis. **b**, Representative micrograph of ClbP embedded in vitreous ice (scale bar = 600 Å; from 3888 micrographs), low pass and high pass filtered for clarity. **c**, Selected 2D class averages of ClbP. **d**, Reconstruction of ClbP filtered and colored by local resolution. Complete data analysis procedure is in Extended Data Fig. 7a. **e**, Gold-standard Fourier shell correlation (FSC) curves from cryoSPARC. **f**, Viewing direction distribution plot. Source data

**Extended Data Fig. 7:**
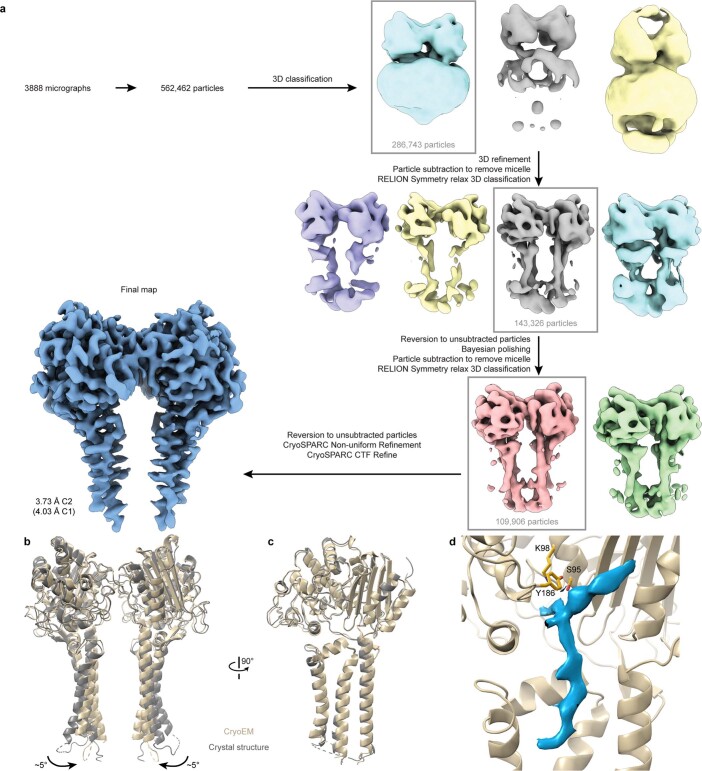
Cryo-EM data processing procedure and model of dimeric ClbP. **a**, Processing scheme for classification and refinement of ClbP. Locally filtered map with dust hidden used for the final reconstruction for clarity. **b**, Superposition of the cryo-EM (tan) and crystal (gray) structures of dimeric ClbP. Both structures are nearly identical (RMSD of 0.830 Å over 904 residues), except for a ~5° bend of the TMDs towards the center of the dimer cavity in the cryo-EM structures. **c**, Superposition of the cryo-EM and crystal structures focusing on a single subunit. View related to **b** by a 90° rotation. **d**, The 3D reconstruction of dimeric ClbP revealed a branched density in the active site that likely corresponds to a copurified lipid. Density is contoured at 5.5σ and the catalytic triad residues are shown as sticks for reference.

To directly probe the oligomeric state of detergent-solubilized ClbP, we determined its structure by single-particle cryo-EM. The two-dimensional (2D) class averages derived from this analysis are consistent with the head-to-head dimer observed in the crystal lattice, and none suggests the presence of a monomeric species (Extended Data Fig. 6a–c). After 2D and 3D classification, we selected 109,906 particles and generated a density map with a nominal resolution of 3.73 Å (Fig. 4d and Extended Data Figs. 6d–f and 7a). The resulting dimeric ClbP structure is nearly identical to the crystallographic dimer (RMSD of 0.830 Å over 904 residues), except for a ~5° bend of each TMD toward the dimer axis (Extended Data Fig. 7b,c). We also identified a branched density in the active site of both subunits that likely corresponds to co-purified phospholipids (Extended Data Fig. 7d). Overall, our cryo-EM structure confirms that ClbP forms a stable dimer in solution through interactions matching those observed in the crystal lattice.

**Extended Data Fig. 8:**
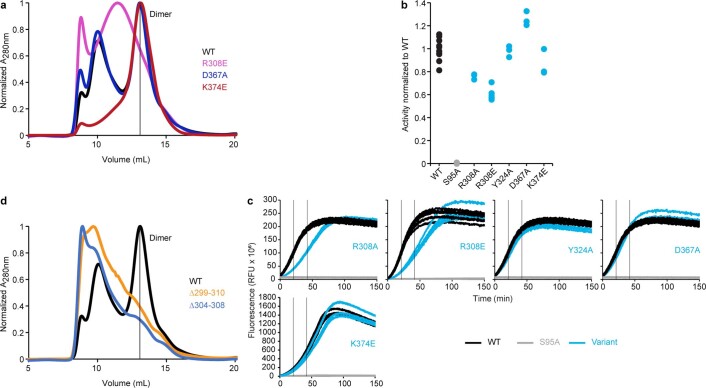
The dimer interface is important for the stability of ClbP. **a**, Superposed normalized size exclusion chromatograms of wild-type ClbP and variants with mutations at the dimer interface performed on a Superdex 200 10/300 column. While no mutation yields a detectable monomeric species, R308E induces the formation of higher molecular weight aggregates. The vertical line indicates the elution volume of dimeric ClbP. **b**, Enzyme activity measurements of dimer interface variants normalized to the wild-type average, using the in vitro fluorogenic activity assay (number of experimental replicates: n = 13 (WT and S95A), 6 (R308E) or 3 (all others)). **c**, Raw fluorescence versus time curves for activity assays performed with dimer interface mutants. Each panel represents replicate measurements (n = 3 for R308A, Y324A, D367A, and K374E and n = 6 for R308E) for the indicated variant (cyan). For comparison, the corresponding replicate measurements for wild-type ClbP (black) and catalytically inactive S95A (gray), measured in the same experiment, are reproduced on each graph. The two gray vertical lines bound the data used for calculating the normalized hydrolysis rates in **b**. **d**, Superposed normalized size exclusion chromatograms of two constructs that replace residues 299-310 or 304-308, respectively, of the longest interface loop with a two-glycine linker. Both constructs elute primarily as higher molecular weight aggregates, suggesting the dimer interface is crucial for the integrity of biochemically isolated ClbP. Source data

We sought to determine the effect of dimer interface mutations R308E, D367A and K374E to the oligomeric state of ClbP by size-exclusion chromatography (SEC). None of these mutations led to the appearance of smaller species, although R308E increased the proportion of aggregates and decreased the proportion of dimers (Extended Data Fig. 8a). In an in vitro assay, these three dimer interface variants, as well as R308A and Y324A, had wild-type-like activity levels for our monomeric fluorogenic probe (Extended Data Fig. 8b,c). Because none of the tested single mutations produced observable monomeric species, we generated two constructs replacing residues 299–310 or 304–308, respectively, of the longest interface loop with a two-glycine linker. Although we could purify these constructs, SEC suggested that they form large aggregates (Extended Data Fig. 8d). Our results suggest that effective disruption of the dimerization interface destabilizes ClbP, and they point to an essential role of this interface in protein stability.

**Extended Data Fig. 9:**
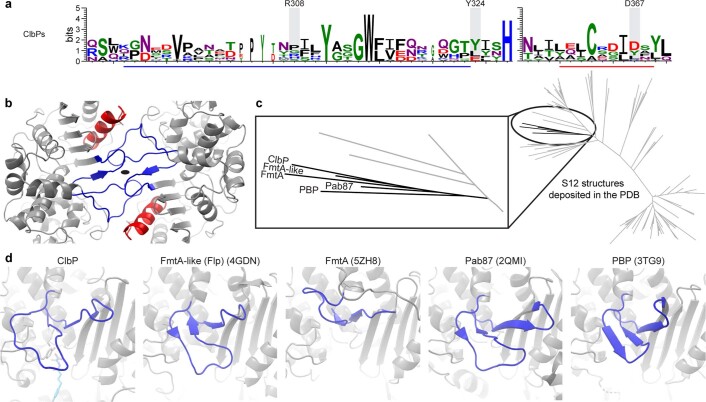
ClbP dimerizes through loops that are not highly conserved. **a**, Sequence logo representing conservation of dimer interface regions highlighted in panel **b** on the structure and in Supplementary Figure 3 on the sequence among 15 ClbP homologs from colibactin biosynthetic clusters. Residues predicted to be important for dimerization are not strongly conserved, suggesting that the mode of dimerization we observe in *E. coli* ClbP may be an adaptation of Proteobacterial ClbP. **b**, ClbP dimer interface highlighting the α8-β11 loop region (residues 296-324; blue) and α11 helix (357-372; red). **c**, Unrooted sequence similarity tree of S12 homologs with structures deposited in the PDB. The inset details the homologs in the same clade as ClbP. **d**, Equivalent views of the α8-β11 loop region (blue) in the structures of homologs in the same clade as ClbP. The α8-β11 loop region are highly variable in structure in the S12 homologs. These regions only mediate formation of a dimer in ClbP and FmtA (PDB ID: 5ZH8), but the dimer geometry is different and only the ClbP dimer has the active sites of the two subunits facing each other on either side of the substrate-binding cavity. The catalytic triad and product analog of ClbP are shown as sticks for context.

The sequences of the two loops forming the ClbP dimer interface are poorly conserved among prodrug-activating peptidases at large or even among ClbP homologs encoded in other colibactin gene clusters (Extended Data Fig. 9a and Supplementary Fig. 3). To determine whether the secondary structures involved in ClbP dimerization are conserved in closely related peptidases, we built a sequence similarity tree of the S12 homologs deposited in the Protein Data Bank (PDB) and compared our structure of ClbP to structures in the same clade. Although the interface loops are present in all the closely related structures, they vary in size and do not mediate the same protein–protein contacts found in the ClbP dimer (Extended Data Fig. 9b–d). Of note, all prodrug-activating ClbP homologs with identified substrates hydrolyze a single prodrug motif on their target substrate (Supplementary Fig. 4). These analyses suggest that dimerization through this interface is a unique and emergent feature of ClbP, which may reflect the pseudodimeric structure of its substrate.

### The ClbP dimer binding site can accommodate precolibactin

To determine whether the substrate-binding site of ClbP can accommodate the proposed structure of precolibactin, we docked precolibactin within the ClbP dimer cavity. Considering its size and symmetry, we divided precolibactin into three overlapping fragments that should recapitulate the interactions formed by the full-length molecule while remaining tractable docking targets (Supplementary Fig. 5a). We used our inhibitor-bound structure^22^ as a template to dock fragments representing the two terminal prodrug motifs to the active site of each ClbP subunit. As expected, the resulting poses for these fragments recapitulate the interactions observed between ClbP and the inhibitor, with the acyl chain in the prodrug motif interacting with W466 and F462 in the TMD and the carbonyl from the scissile amide bond 3.6 Å away from the S95 γO. We then performed unrestrained docking of the central precolibactin fragment to the intersubunit interface. Out of several possible poses bridging the two subunits, we selected one in which the terminal functional groups roughly overlap with the corresponding groups in the two end fragments in their chosen poses (Supplementary Fig. 5b,c). Finally, we generated a model of precolibactin docked in the dimer cavity by manually aligning the shared groups of the separately docked fragments and performing a global minimization in the assembled precolibactin (Fig. 4e,f). Overall, our model illustrates that the ClbP dimer can accommodate the proposed full-length precolibactin structure and optimally position the two prodrug motifs for simultaneous activation.

## Discussion

Colibactin is an unusual natural product with important implications for human health, yet determining its chemical structure remains challenging. The proposed pseudodimeric colibactin structure explains aspects of its bioactivity—such as the ability to introduce inter-strand cross-links in DNA—and requires the activity of all biosynthetic enzymes in the *pks* island. In this study, we investigated the mechanism underlying the conversion of precolibactin to the active colibactin genotoxin by ClbP. Our study details how ClbP recognizes and cleaves the *N*-acyl-d-asparagine prodrug motif from precolibactin, addressing the role of the TMD in enzymatic function and the basis of substrate specificity. We discovered that ClbP forms a dimer that may promote the simultaneous activation of the two colibactin warheads, further supporting the proposed pseudodimeric structure of precolibactin.

The requirement of the full ClbP TMD for enzyme activity^21^ is explained by the substrate interactions with the TM2–TM3 linker. W466 contacts the acyl group of the prodrug motif, and the W466A mutation inactivates ClbP. Beyond its roles in substrate binding, the ClbP TMD may enable interactions with other proteins, such as the precolibactin transporter ClbM or, as recently suggested, the MchF microcin exporter^30^. Besides W466, the only TMD residue strictly conserved among prodrug-activating peptidases is W460, yet the W460A mutation did not affect cleavage of our monomeric probe. The region around W460—in the TM2–TM3 linker with its sidechain wedged between TM2 and TM3—is a good candidate for mediating contacts with other proteins.

Hydrogen bonds from S188 and N331 to the prodrug d-asparagine sidechain are crucial for activity, and N331 is critical for d-asparagine specificity. The severe functional impairment by S188A and N331A, combined with the inability of ClbP to process substrates with d-alanine prodrug motifs^20^, suggest that d-asparagine–ClbP interactions are essential to bind and orient the substrate for catalysis. Conversely, the H257A mutation had little effect on peptidase activity, suggesting that the d-asparagine–H257 interactions are less important for prodrug cleavage. Accordingly, the OH–O angle between the S188 hydroxyl and the substrate’s carbonyl acceptor is 175°, more optimal for hydrogen bonding than the 124° NH–O angle formed with H257. Furthermore, position 257 is not conserved among prodrug-activating peptidases and predominantly corresponds to asparagine. A H257N substitution would likely place hydrogen-bonding groups too far to strongly interact with the substrate’s d-asparagine sidechain carbonyl. The N331 sidechain carbonyl enforces substrate specificity by selecting for prodrug motifs containing d-amino acids with hydrogen-bond donors that can interact with it, explaining why ClbP does not cleave d-aspartate motifs. Accordingly, the E92Q mutation, which disrupts the interaction network that stabilizes and orients N331, substantially increased cleavage of a d-aspartate-containing substrate. Because E92Q also cleaves d-asparagine substrates at a wild-type-like rate, the E92Q mutation likely results in an N331 sidechain without preferential orientation that can either accept hydrogen bonds from the d-asparagine amide NH or donate hydrogen bonds to the carboxylate in d-aspartate. N331 is strictly conserved among all prodrug-activating peptidases, suggesting that this mechanism for d-asparagine specificity is also conserved.

The dimer interface that we observed seems unique to Proteobacterial ClbPs and is not conserved in the sequences of other prodrug-activating peptidases or homologs from the broader S12 family. Precolibactin is the only prodrug-activating peptidase substrate proposed to have two prodrug motifs (Fig. 1 and Supplementary Fig. 4), so ClbP may have evolved a dimeric binding site to accommodate it. Colibactin activation involves the formation of reactive electrophiles that must remain intact until they encounter DNA and produce the inter-strand cross-links that characterize colibactin toxicity. Dimeric ClbP could, thus, be important to simultaneously activate the two warheads and ensure that most of the colibactin produced has two active warheads. Because inter-strand cross-links and the resultant double-strand breaks are especially deleterious, the synthesis of toxins that inflict this type of DNA damage on other organisms may offer a competitive advantage to colibactin-producing bacteria^31,32^. The functional role of the ClbP dimer and the effects of its disruption on colibactin maturation remain unknown. Our results indicate the dimer interface is crucial for ClbP stability and, therefore, suggest that the role of dimerization cannot readily be studied with dimer-disrupting mutations. A previous study found that mutation of D367, which forms a buried ion pair with R308 at the dimer interface, to leucine impairs colibactin maturation by ClbP (ref. ^21^). The authors concluded that this was due to a disruption of the negative electrostatic surface potential. Considering the dimeric ClbP structure, this mutation may, instead, impair ClbP dimerization or stability and, in turn, reduce its ability to produce colibactins capable of causing double-strand breaks. Disrupting the ClbP dimer could potentially lead to the production of aberrant colibactins that are still genotoxic, albeit by inflicting different DNA lesions. Finally, the ClbP dimer cavity may play a role in protecting the warheads of mature colibactin from reacting with periplasmic nucleophiles before being exported from the producing cell.

Intact colibactin has never been isolated, so its structure has been inferred based on biosynthetic pathway intermediates and the enzymes that assemble them and total synthesis of a candidate molecule^8^. Several structures that explain observations about the biochemistry and activity of colibactin have been proposed, with the currently favored pseudodimeric structures explaining the characteristic cross-linking ability of colibactin. The ClbP dimer demonstrated here supports the biological relevance of a pseudodimeric candidate precolibactin and offers clues into potential mechanisms to regulate toxin activation and preserve the reactivity of its warheads toward DNA.

## Methods

### Constructs

ClbP constructs were derived from a previously described plasmid (Addgene, 48244)^15^ containing the *Escherichia coli* CFT073 *clbP* sequence (GenBank ID: NP_754344.1) inserted between the NdeI and XhoI sites of pET29b. Constructs used for crystallization had the pET29b-encoded C-terminal 6×His tag, whereas constructs used in functional assays, SEC experiments and cryo-EM had a C-terminal 10×His tag introduced by extending the 6×His tag through site-directed mutagenesis. Mutations were introduced using the QuikChange mutagenesis protocol (Agilent) and confirmed by Sanger DNA sequencing of the whole open reading frame. Mutagenesis primers are listed in Supplementary Table 2.

### Protein expression

Single colonies of transformed C41(DE3) (Lucigen) were inoculated into lysogeny broth and shaken for 7 hours at 37 °C. Terrific Broth with 50 µg ml^−1^ of kanamycin was inoculated 1:100 with starter culture and shaken at 37 °C until the optical density at 600 nm (OD_600_) reached 0.3 and then was transferred to 15 °C and grown until OD_600_ ~0.5–0.6. Cultures were induced with 0.5 mM isopropyl β-d-1-thiogalactopyranoside and incubated at 15 °C for 20 hours. Cells were harvested by centrifugation at 4,544*g* for 15 minutes and flash-frozen in liquid nitrogen. The selenomethionine-substituted protein was expressed using a protocol for suppression of endogenous methionine synthesis^33^ and L-selenomethionine (Anatrace).

### Protein purification for crystallography

All steps were performed at 4 °C. Cells were resuspended in load buffer (20 mM sodium phosphate pH 8.0, 20 mM imidazole, 500 mM NaCl and 10% glycerol) with 1 mM phenylmethane sulfonyl fluoride and 1 mM benzamidine and lysed by sonication on ice (six 45-second cycles with a Branson Sonifier 450 under 65% duty cycle and output control of 10). Lysates were cleared by centrifugation at 48,300*g* for 20 minutes. Membranes were pelleted by ultracentrifugation at 235,000*g* for 70 minutes, resuspended in load buffer, homogenized using a glass Potter–Elvehjem grinder and solubilized in 1% (w/v) *n*-dodecyl-*β*-d-maltoside (DDM, Anatrace) while mixing for 2 hours. Detergent-insoluble materials were precipitated by ultracentrifugation at 142,000*g* for 35 minutes, and the supernatant was incubated with pre-equilibrated Ni-Sepharose resin (Qiagen) while mixing for 2 hours. The resin was washed in (1) 12 column volumes (CV) of load buffer containing 0.03% DDM; (2) 10 CV of load buffer containing 0.5% lauryl maltose neopentyl glycol (LMNG, Anatrace); and (3) 12 CV of load buffer containing 0.1% LMNG. ClbP was eluted with 9 CV of load buffer containing 450 mM imidazole and 0.01% LMNG and further purified by SEC on a Superdex S200 10/300 column (GE Healthcare) equilibrated with 10 mM Tris-HCl pH 8.1, 150 mM NaCl and 0.003% LMNG. The selenomethionine-substituted protein was purified similarly, except the Ni-affinity resin wash and elution and SEC buffers were supplemented with 1 mM DTT. ClbP-rich SEC fractions were pooled and concentrated to 24 mg ml^−1^ using a 50-kDa molecular weight cutoff (MWCO) centrifugal filter (EMD Millipore), flash-frozen in liquid nitrogen and stored at −80 °C.

### Protein purification for functional assays and SEC

Proteins used in functional assays and SEC were purified as above, except that the load buffer contained 55 mM imidazole, and all washes contained 0.05% DDM. Protein used in functional assays was eluted from the Ni-Sepharose resin in a stepwise imidazole gradient of 2-CV fractions containing 75, 100, 125, 150, 200 and 450 mM imidazole. The 200-mM and 450-mM imidazole elutions were pooled and dialyzed overnight in 3.5-kDa MWCO tubing (Thermo Fisher Scientific) against 50 mM Tris-HCl pH 8.0, 200 mM NaCl and 0.02% DDM and then concentrated using a 100-kDa MWCO centrifugal filter (EMD Millipore) to minimize concentration of empty detergent micelles and flash-frozen. Proteins used for SEC were eluted with steps of 75, 100, 150, 250, 300 and 450 mM imidazole. The 250-mM and 300-mM imidazole elutions were pooled and concentrated in a 50-kDa MWCO centrifugal filter for loading onto the SEC column.

### ClbP crystallization

ClbP was reconstituted in monoolein or monopalmitolein mesophases (protein-to-lipid volume ratio of 1:1.5 and 1:1 respectively) using the syringe reconstitution method. The protein bolus was dispensed onto custom-made 96-well glass sandwich plates in 75-nl drops using an NT8 drop-setting robot (Formulatrix) and overlaid with 900 nl of precipitant. The precipitant for monoolein-bound crystals was a mixture of 200 nl of precipitant 1 (0.1 M imidazole pH 7.8, 10% (v/v) PEG400, 150 mM Li_2_SO_4_, 5.5 mM *N*-4-(4-bromophenyl)butanoyl-d-asparaginyl-l-alanine methyl ester (compound **1**)) and 700 nl of precipitant 2 (0.1 M Tris-HCl pH 7.4, 28% (v/v) PEG400, 100 mM Li_2_SO_4_ and 4% (v/v) polypropylene glycol). For product-bound crystals, 1.2% (w/v) myo-inositol replaced the polypropylene glycol in precipitant 2. The monoolein-bound structure was obtained from crystals in which the substrate analog was supplemented only in the precipitant. The product-bound structure was obtained from crystals grown in monopalmitolein and that were supplemented with the substrate analog directly in the lipid (excess compound was added to the molten lipid, and undissolved solids were separated by centrifugation) and in the precipitant. Crystals appeared within 1–2 days, reached their optimal size after 9 days and were harvested after 12–30 days using mesh loops (MiTeGen) and plunged into liquid nitrogen.

### Diffraction data collection and processing

Diffraction data for the monoolein-bound and product-bound structures were collected at beamline 24ID-C of the Advanced Photon Source at wavelengths of 0.98 Å and 0.92 Å, respectively. Datasets from 1 (monoolein-bound structure) or 2 (product-bound structure) crystals were indexed in XDS^34^, scaled in CCP4 AIMLESS^35^ and phased by molecular replacement in PHENIX^36^ using the structure of the periplasmic domain as search model (PDB ID: 3O3V, chain A)^19^. Data statistics are in Supplementary Table 1.

### Refinement and model building

Model building was done in Coot^37^ and refinement in PHENIX, with macrocycles including reciprocal space refinement, individual B-factors, TLS groups and optimization of the X-ray/ADP weights. Ligand restraints were generated in Phenix.elbow with automatic geometry optimization. To address prominent negative density centered around the bromine atoms of our structure in presence of compound **1**, the bromines were defined as an ‘anomalous group’ with the reference f′ and f′ values (−8.5385 and 3.8222, respectively) suggested by Phenix.form.factor. Remaining negative density not accounted for by the anomalous scattering of the bromines was addressed by refining the occupancy of the individual bromine atoms. Photodissociation of bromines from brominated molecules in crystal structures collected at the bromine absorption edge was described previously in other structures^38^. The crystal-packing interactions involve extensive contacts between periplasmic domains of symmetry mates and limited contacts between the TMD and the periplasmic domains in stacked layers along the *c* axis. Accordingly, the electron density map quality in the TMD is variable, with regions closest to the periplasm better resolved than regions closer to the cytoplasm (Supplementary Fig. 1b). To model the intracellular ends of transmembrane helices, the map blurring feature in Coot was used to accentuate low-resolution features in our 2F_o_–F_c_ map and build the backbone corresponding to residues 411–418 and 492–496. The final model of product-bound ClbP includes residues 36–418 and 427–496; 96.21% of backbone atoms are in Ramachandran favored regions, 3.79% in allowed regions, with no outliers. The final model of monoolein-bound ClbP includes residues 35–414 and 430–495; 96.61% of backbone atoms are in Ramachandran favored regions, 3.39% in allowed regions, with no outliers. Model statistics are listed in Supplementary Table 1.

Selenium and bromine anomalous difference Fourier maps were generated in phenix.maps using 4-Å and 4.5-Å high-resolution cutoffs, respectively. Polder maps were generated in phenix.polder using a solvent exclusion radius of 5.0 and a resolution factor of 0.25.

Structural biology applications used in this project were compiled and configured by SBGrid^39^.

### Cryo-EM sample preparation

ClbP was purified by Ni-affinity chromatography as above, except that protein was eluted with a stepwise gradient of 75, 100, 150, 250, 300 and 450 mM imidazole. To minimize the amount of aggregated protein, only the 250-mM and 300-mM fractions were pooled, concentrated and then loaded onto a Superdex S200 10/300 column (GE Healthcare) equilibrated in 10 mM HEPES pH 7.3, 200 mM NaCl and 0.06% GDN (glyco-diosgenin, Anatrace). The peak fraction (3.5 mg ml^−1^) was used for cryo-EM analysis (Extended Data Fig. 6a): 3 µl of sample was deposited onto 400 mesh QUANTIFOIL Cu 1.2/1.3 grids that had been glow-discharged in a PELCO easiGLOW (Ted Pella) at 0.39 mBar, 15 mA for 30 seconds. Samples were vitrified in 100% liquid ethane using a Vitrobot Mark IV (Thermo Fisher Scientific), with a wait time of 30 seconds, a blot time of 5 seconds and a blot force of 16 at 100% humidity.

### Cryo-EM data collection and processing

Cryo-EM data were collected on a 300-kV Titan Krios G3i Microscope (Thermo Fisher Scientific) equipped with a K3 direct electron detector (Gatan) and a GIF quantum energy filter (20 eV) (Gatan) using counted mode at the Harvard Cryo-Electron Microscopy Center for Structural Biology at Harvard Medical School. Data were acquired using image shift and real-time coma correction by beamtilt using the automated data collection software SerialEM^40^; nine holes were visited per stage position, acquiring two movies per hole. Details of the data collection and dataset parameters are summarized in Supplementary Table 3. Dose-fractionated images were gain-normalized, aligned, dose-weighted and summed using MotionCor2 (ref. ^41^). Contrast transfer function (CTF) and defocus value estimation were performed using CTFFIND4 (ref. ^42^). Details of the data processing strategy are in Extended Data Fig. 7a. In short, particle picking was carried out using crYOLO^43^, followed by initial 3D classification within RELION^44^, yielding 286,743 particles. To improve classification, the micelle was removed via particle subtraction followed by RELION symmetry relax 3D classification. The particles in the best-looking class (143,326) were selected, reverted to unsubtracted particles, 3D refined and Bayesian polished. After Bayesian polishing, a subsequent round of particle subtraction/RELION symmetry relax 3D classification was performed. The best class (109,906) was selected as the final dataset. After reversion to unsubtracted particles, the data were subjected to CTF refinement and non-uniform refinement with C2 symmetry imposed, in cryosSPARC^45^, to produce the final 3.73-Å reconstruction (4.03 Å C1).

### Cryo-EM model building and refinement

A model of dimeric ClbP generated from the asymmetric unit in 7MDF was built onto the C2 symmetrical map using Coot. Refinement was done in Phenix.real_space_refine with macrocycles including morphing, global minimization, local grid search and ADP, under secondary structure and non-crystallographic symmetry (NCS) constraints. Each round of refinement was followed with inspection of each chain and remodeling into the density by simulations run in ISOLDE^46^.

### Docking

All docking experiments were performed with Flare version 3.0.0 (Cresset). Because precolibactin is large and the ClbP dimer cavity provides an extensive binding surface, precolibactin was divided into three fragments that were docked to different sections of the protein. Two fragments represented the precolibactin termini and extended from the prodrug motif to the cyclopropane. The third fragment represented the precolibactin center, encompassing the two thiazoles and overlapping with the two termini. The fragments representing the precolibactin termini (prodrug motif simplified to hexanoyl-d-asparagine) were docked to the active site of each subunit in a grid defined by the residues that interact with substrate analogs in our structures, using the inhibitor in the inhibitor-bound structure^22^ as a template. The calculation was done by the ‘Very Accurate but Slow’ method, ‘Extra Precision’ quality, with the maximum number of poses set to 100. The top-scoring poses from docking the termini extended the molecule toward the dimer center, so the central precolibactin fragment was docked in a rectangular grid defined by residues S95, D306, I309 and W466 from both subunits using no template and under identical parameters. Full precolibactin was modeled in the ClbP dimer by aligning the chosen poses of the three individually docked fragments and manually rotating and drawing bonds between overlapping moieties. The assembled molecule was globally minimized in Flare using the ‘Accurate’ calculation method.

### Synthesis of substrate analogs

The ClbP fluorogenic probe and substrates used for activity assays were synthesized as described^20^. See the Supplementary Note in the Supplementary Information for synthetic protocols and spectral data for compound **1**.

### Fluorescence-based activity assay

Activity assays with purified protein were performed in black 384-well square flat-bottom plates as described^20^. Each reaction contained 100 nM ClbP and 50 µM fluorogenic probe in 25 µl of 50 mM Tris-HCl pH 8.0, 200 mM NaCl and 0.02% DDM. All reactions within a replicate set were initiated simultaneously by the addition of ClbP to a probe-containing master mix. Hydroxycoumarin fluorescence (excitation: 340 nm, emission: 442 nm) was measured every 30 seconds for 3 hours in a SpectraMax i3 plate reader (Molecular Devices). The enzymatic activity of each sample was estimated as the slope of the linear fit of data recorded between 21 minutes and 42 minutes, and the relative activity was calculated with respect to the average for the wild-type triplicates.

### LC–MS-based activity assay with dipeptide substrates

Endpoint assays measuring cleavage of dipeptide substrates with alternative prodrug motifs by ClbP variants were set up in a 96-well plate as described^20^. Enzyme solutions were prepared by diluting purified ClbP variants to 600 nM in assay buffer. The different dipeptide substrates were prepared by diluting 10 mM stocks (in DMSO) to 120 µM in assay buffer. All reactions were initiated simultaneously upon mixing 25 µl of enzyme solution with 125 µl of the respective substrate (final concentration of 100 nM enzyme, 100 μM substrate and 1% DMSO) and incubated for 5 hours at 25 °C. Reactions were quenched and prepared for LC–MS analysis by adding 20 µl of reaction mixture to 180 µl of cold methanol. LC–MS analysis was performed on an Agilent 6530 Q-TOF Mass Spectrometer fitted with a dual-spray electrospray ionization (ESI) source. The capillary voltage was set to 3.5 kV, the fragmentor voltage to 175 V, the skimmer voltage to 65 V and the Oct 1 RF to 750 V. The drying gas temperature was maintained at 275 °C with an 8 L min^−1^ flow rate and a nebulizer pressure of 35 psi. A standard calibrant mix was introduced continuously during all experiments via the dual-spray ESI source. Chromatography was performed using an Agilent 1200 series LC on a Hypersil GOLD aQ C18 reverse phase column (50 ×3 mm, Thermo Fisher Scientific) with the following elution conditions: a gradient from 35% A: 65% B to 100% A over 5 minutes, holding at 100% A for 2 minutes, followed by a gradient back to 35 % A over 1 minute and holding at 35% A for 3.5 minutes (solvent A: acetonitrile + 0.1% formic acid; solvent B: water + 0.1% formic acid; flow rate = 0.4 ml min^−1^; injection volume = 10 µl). All experiments were performed in positive ion mode, and the masses detected corresponded to [M+H]^+^ ions. To compare relative conversion of different substrates, the extracted ion chromatogram (EIC) for each substrate was analyzed using the Quantitative Analysis software platform (Agilent) to determine an area under the curve (AUC) for each compound whose mass and retention time were compared against a synthetic standard.

### Sequence analyses

Sequences corresponding to all members of the β-lactamase superfamily (PF00144) were downloaded from the UniProt database; sequences corresponding to UniRef90 members were also downloaded. All sequences smaller than 400 or larger than 1,200 amino acids, or with fewer than two transmembrane helices (as predicted by TMHMM), were trimmed from the dataset. These sequences were supplemented by tblastn searches against the National Center of Biotechnology Information (NCBI) NR and WGS databases (excluding all matches from the genera *Citrobacter*, *Enterobacter*, *Escherichia*, *Klebsiella* and *Salmonella*) using the *E. coli* CFT073, *Pseudovibrio denitrificans* DSM 17465 and *Paenibacillis donghaensis* KCTC 13409 ClbP homologs. In some cases, sequences were identified in unannotated scaffolds; in these cases, the scaffolds were annotated via RASTtk, and annotations were supplemented with InterProScan^47^ for all predicted coding sequences.

Genome neighborhoods were obtained by procuring the source genomes associated with the UniProt records from the European Nucleotide Archive (ENA) or NCBI databases. Scaffolds lacking homologs were removed, and genes in the immediate genomic neighborhood (±10 genes) of the PF00144-encoding gene were re-annotated using InterProScan to standardize annotation. PF00144 family members from truncated neighborhoods (with <10 genes between the gene of interest and the end of the scaffold) were excluded from further analyses. Comparison of genomic neighborhoods to characterized biosynthetic gene clusters permitted the classification of gene clusters as *ami*-like, *clb*-like, *ede*-like, *pam*-like, *xcn*-like and *zma*-like, along with the identification of genomic regions encoding predicted but unidentified natural products (Supplementary Fig. 2). For genome clusters of particular interest, including the *ede* cluster and several *clb*-like clusters in *Pseudovibrio* and *Paenibacillus* strains, this included natural product-focused analysis to confirm gene cluster identification with PKS/NRPS Analysis^48^, antiSMASH^49^ and PRISM^50^.

Sequences of the remaining PF00144 homologs from UniProt and NCBI were pooled and submitted to the EFI-EST web server^51^. Representative nodes were chosen at 100% ID to eliminate identical sequences (yielding 730 representative nodes) and an expectation value cutoff of 1 × 10^−90^ was used for initial visualization in Cytoscape^52^ (Extended Data Fig. 1a–d). Unless otherwise specified, representative node sequences were used for analyses. To exclude PF00144 homologs unlikely to be members of a ClbN/ClbP-like prodrug synthesis/peptidase system, family members that were in the genomic neighborhood of a gene encoding a condensation and adenylation domain were identified. Four clusters of sequences within the SSN—overlapping entirely with the clusters in which known prodrug peptidases are found—were predominantly composed of sequences from genomic neighborhoods including NRPS machinery, and these sequences were used for investigations using the larger prodrug peptidase family. Sequences of representative nodes from ClbPs only, from all probable prodrug peptidases and from all SSN components were aligned using Clustal Omega, and sequence logos were generated in WebLogo 3.

To build a tree of ClbP homologs with available structures, jackHMMER^53^ was used to query the PDB with the seed alignment of the protein superfamily (PF00144) downloaded from PFAM. To remove duplicate or near-duplicate sequences, the 1,365 sequences from the output were clustered (99% identity threshold) in the CD-HIT webserver. The 153 sequence representatives of each cluster were aligned using Clustal Omega, and a maximum likelihood tree was built from this alignment in PhyML using the default parameters and automatic model selection by SMS^54^. Analysis and rendering of the tree was done in Archeopteryx^55^. Sequence logos for the broader S12 family were created in WebLogo 3 using an alignment of the 901 sequences in the S12 library alignment downloaded from the MEROPS database^18^.

### Reporting summary

Further information on research design is available in the Nature Research Reporting Summary linked to this article.

## Online content

Any methods, additional references, Nature Research reporting summaries, source data, extended data, supplementary information, acknowledgements, peer review information; details of author contributions and competing interests; and statements of data and code availability are available at 10.1038/s41589-022-01142-z.

## Supplementary information

Supplementary Information Supplementary Tables 1–3, Supplementary Figs. 1–5 and Supplementary Note

Reporting Summary

Supplementary Data Data for sequence analyses

## Data Availability

Atomic coordinates and structure factors for the reported crystal structures in this work have been deposited to the Protein Data Bank under accession numbers 7MDE (Monoolein-bound S95A-L454M-I478M (SeMet) ClbP) and 7MDF (Product-bound S95A-L454M-I478M ClbP). Corresponding X-ray diffraction images have been deposited to the SBGrid Data Bank under accession numbers 833 (doi:10.15785/SBGRID/833) and 831 (doi:10.15785/SBGRID/831), respectively. The map of the cryo-EM reconstruction has been deposited to the Electron Microscopy Data Bank (accession number: EMD-26593) and the refined coordinates to the PDB (ID: 7UL6). The sequences for bioinformatic analyses were procured from PFAM (seed alignment version 33.1), UniProt (2021_02 release), GenBank (release 242), ENA (2021.03.03) and MEROPS (12.4), and the dataset (SSN, aligned sequences and phylogenetic tree) is in the Supplementary Data. Source data for Figs. 2 and 3 and Extended Data Figs. 3, 4, 6 and 8 are provided with this paper.

## Source data

Source Data Fig. 2 Statistical Source Data
Source Data Fig. 3 Statistical Source Data
Source Data Extended Data Fig. 3 Statistical Source Data
Source Data Extended Data Fig. 4 Statistical Source Data
Source Data Extended Data Fig. 6 Statistical Source Data
Source Data Extended Data Fig. 8 Statistical Source Data
