# Supplementary material for: Structural basis of colibactin activation by the ClbP peptidase
Source: Nat Chem Biol. 2022 Oct 17;19(2):151–8. doi: 10.1038/s41589-022-01142-z (PMC9889268; doi:10.1038/s41589-022-01142-z)
Supplement: Supplementary file 1 — Supplementary Tables 1–3, Supplementary Figs. 1–5 and Supplementary Note [file 41589_2022_1142_MOESM1_ESM.pdf]

nature chemical biology

Article

https://doi.org/10.1038/s41589-022-01142-z

Structural basis of colibactin activation by the ClbP peptidase

In the format provided by the authors and unedited

Supplementary Information

Structural basis of colibactin activation by the ClbP peptidase

José A. Velilla[1], Matthew R. Volpe[2], Grace E. Kenney[2], Richard M. Walsh Jr[3,4], Emily P. Balskus[2,5], and Rachelle Gaudet[1]

Affiliations

1 Department of Molecular and Cellular Biology, Harvard University, Cambridge, MA USA

2 Department of Chemistry and Chemical Biology, Harvard University, Cambridge, MA USA

3 The Harvard Cryo-EM Center for Structural Biology, Harvard Medical School, Boston, MA

4 Department of Biological Chemistry and Molecular Pharmacology, Blavatnik Institute, Harvard Medical School, Boston, MA

5 Howard Hughes Medical Institute, Harvard University, Cambridge, MA USA

This file contains:

- Supplementary Table 1. Data collection and refinement statistics (molecular replacement)
- Supplementary Table 2. List of primers used for site-directed mutagenesis
- Supplementary Table 3. Cryo-EM data collection, refinement, and validation statistics
- Supplementary Figure 1. Structure of full-length ClbP
- Supplementary Figure 2. Sample biosynthetic gene clusters
- Supplementary Figure 3. Alignment of representative homologs
- Supplementary Figure 4. ClbP is the only prodrug-activating peptidase proposed to process a pseudodimeric substrate
- Supplementary Figure 5. Docking experiments to model precolibactin binding to ClbP
- Supplementary Note (Synthesis and validation of compound 1)

Additional Supplementary Data provided separately is a zip file containing:

- README.txt file
- Sequence similarity network for PF00144 family members in the correct size range and with at least two transmembrane domains (.cys file)
- Metadata file for sequences in the sequence similarity network (.xlsx file)
- Clustal Omega alignment for sequences from all representative nodes (.fa file)
- Clustal Omega alignment for sequences from all prodrug peptidase representative nodes (.fa file)
- Clustal Omega alignment for sequences from all ClbP representative nodes (.fa file)
- Clustal Omega alignment for all S12 sequences used in phylogenetic analyses (.fa file)
- Phylogenetic tree for S12 family members with available structures (.nwk file)

Supplementary Table 1. **Data collection and refinement statistics (molecular replacement)**

	Product-bound S95A-L454M-I478M (PDB: 7MDF)	Monoolein-bound S95A-L454M-I478M (SeMet) (PDB: 7MDE)
**Data collection**		
Space group	P 4_2 2_1 2	P 4_2 2_1 2
Cell dimensions		
a, *b*, *c* (Å)	96.69, 96.69, 182.74	97.47, 97.47, 183.97
α, β, γ (°)	90, 90, 90	90, 90, 90
Resolution (Å)	46.74 - 2.3 (2.38 - 2.3)	48.74 - 2.7 (2.8 - 2.7)
Total reflections	673835 (61048)	163298 (16722)
Unique reflections	39268 (3829)	25099 (2449)
$I / \sigma I$	8.89 (1.32)	7.74 (1.22)
R_{sym} or R_{merge}	0.362 (2.781)	0.211 (1.338)
R_{meas}	0.3731 (2.872)	0.2294 (1.447)
CC1/2	0.997 (0.697)	0.994 (0.67)
Completeness (%)	99.81 (99.63)	99.79 (99.96)
Redundancy	17.2 (15.9)	6.5 (6.8)
Wilson B-factor	33.75	48.7
**Refinement**		
Resolution (Å)	46.74 - 2.3 (2.38 - 2.3)	48.74 - 2.7 (2.8 - 2.7)
No. reflections	39236 (3819)	25074 (2448)
No. reflections in R_{free}	1964 (190)	751 (74)
R_{work} / R_{free}	0.1914 / 0.2190	0.1948 / 0.2350
No. atoms	3935	3605
Protein	3431	3341
Ligand/ion	195	86
Water	309	178
B-factors		
Protein	56.56	67.5
Ligand/ion	83.66	76.18
Water	50.09	56.67
R.m.s. deviations		
Bond lengths (Å)	0.007	0.007
Bond angles (°)	0.89	0.92
Ramachandran plot		
Favored (%)	96.21	96.61
Allowed (%)	3.79	3.39
Disallowed (%)	0	0
Rotamer outliers (%)	2.59	2.98
Clashscore	8.33	4.85

Values in parentheses are for highest-resolution shell. Data for the product-bound structure merge reflections from 2 crystals. Data for the monoolein-bound structure were obtained from a single crystal.

Supplementary table 2. **List of primers used for site-directed mutagenesis**

Primer Name	Sequence
**ClbP Add His FWD**	CCACCATCACCATCACTGAGATCCGGCTGCTAACAAAGCCCGAAAG
**ClbP Add His REV**	CTCAGTGATGGTGATGGTGGTGGTGGTGGTGGTGCTCGAGCTC
**E92Q FWD**	GTTTACCAGCTGGGATCGATGAGTAAGGCGTTTAC
**E92Q REV**	CAGCTGGTAAACTGTGTCTAGAGTATTCGCTTTCTGAC
**S95A FWD**	GGGAGCGATGAGTAAGGCGTTTACCGGACTTG
**S95A REV**	CATCGCTCCCAGCTCGTAAACTGTGTCTAGAG
**S188A FWD**	CTATGCCGCCGCCAATTATGATGTGTTGGGCG
**S188A REV**	GCGGCGGCATAGCTAAACTTCGCACCCG
**S188N FWD**	CTATGCCAACGCCAATTATGATGTGTTGGGCGCGGTG
**S188N REV**	GGCGTTGGCATAGCTAAACTTCGCACCCGGCG
**K240A FWD**	CTATGCACTGGGATTCGGCAAACCCGTTCTGTTTCATGC
**K240A REV**	CCAGTGCATAGCCGCTTGCCTTGTTGACAATAATCTCATCC
**F243A FWD**	CTGGGAGCCGGCAAACCCGTTCTGTTTCATGCG
**F243A REV**	TGCCGGCTCCCAGTTTATAGCCGCTTGCCTTGTTGAC
**H257A FWD**	GGAACGCTGTTCCTGCCGCCTATATCCATAGCACTC
**H257A REV**	GAACAGCGTTCCGGGCCAGAGGCGCATGAAAC
**R308A FWD**	GACAATGCTATCCTCTATGCCAGCGGTTGGTTTATCGACCAG
**R308A REV**	GAGGATAGCATTGTCTGCGGCAAGCGGAACATCACTATTACC
**R308E FWD**	CAATGAGATCCTCTATGCCAGCGGTTGGTTTATCGACCAGAATC
**R308E REV**	GAGGATCTCATTGTCTGCGGCAAGCGGAACATCACTATTACC
**N331A FWD**	GGGCAGGCTCCAAACTTTTCTTCTTGCATTGCGTTGCG
**N331A REV**	GTTTGGAGCCTGCCCACCGTGACTGATGTAAGGG
**Y324A FWD**	GCCCTGCCATCAGTCACGGTGGGCAGAATCCAAAC
**Y324A REV**	CTGATGGCAGGGCCTTGATTCTGGTCGATAAACCAACCG
**D367A FWD**	GGATATCGCTAATTATCTGCGCATTGGCAAATATGCTGAC
**D367A REV**	GATAATTAGCGATATCCGCGCAAAGCTGTAGTATCAGATTC
**K374E FWD**	GGCGAATATGCTGACGGCGCTGGTGATGCAATTAC
**K374E REV**	CCGTCAGCATATTCGCCAATGCGCAGATAATTATCGATATC
**W460A FWD**	CTTGACGCGCGTTTTATCTTGGTATGGGGTCCATCGAG
**W460A REV**	AACGCGCGTCAAGTCCTGGAGATAGTATACCCGGTGC
**F462A FWD**	GGCGTGCTATCTTGGTATGGGGTCCATCGAGCG
**F462A REV**	CAAGATAGCACGCCAGTCAAGTCCTGGAGATAGTATACC
**W466A FWD**	GGTAGCGGGTCCATCGAGCGTGTTGGCGATAC
**W466A REV**	GGACCCGCTACCAAGATAAAACGCCAGTCAAGTCCTGG

Supplementary Table 3. Cryo-EM data collection, refinement, and validation statistics

	WT ClbP (EMD-26593) (PDB: 7UL6)
**Data collection and processing**	
Magnification	60606
Voltage (kV)	300
Electron exposure (e–/Å^2)	76.191
Defocus range (μm)	0.8, 2.2
Pixel size (Å)	0.825
Symmetry imposed	C2
Initial particle images (no.)	562462
Final particle images (no.)	109906
Map resolution (Å)	3.73
FSC threshold	0.143
Map resolution range (Å)	3.20 – 5.53
**Refinement**	
Initial model used (PDB code)	7MDF
Model resolution (Å)	4.0
FSC threshold	0.5
Map sharpening *B* factor (Å^2)	188.1
Model composition	
Non-hydrogen atoms	6654
Protein residues	878
B factors (Å^2)	
Protein	58.0
Ligand	N/A
R.m.s. deviations	
Bond lengths (Å)	0.003
Bond angles (°)	0.615
Validation	
MolProbity score	1.32
Clashscore	5.13
Poor rotamers (%)	0.15
Ramachandran plot	
Favored (%)	97.81
Allowed (%)	2.19
Disallowed (%)	0

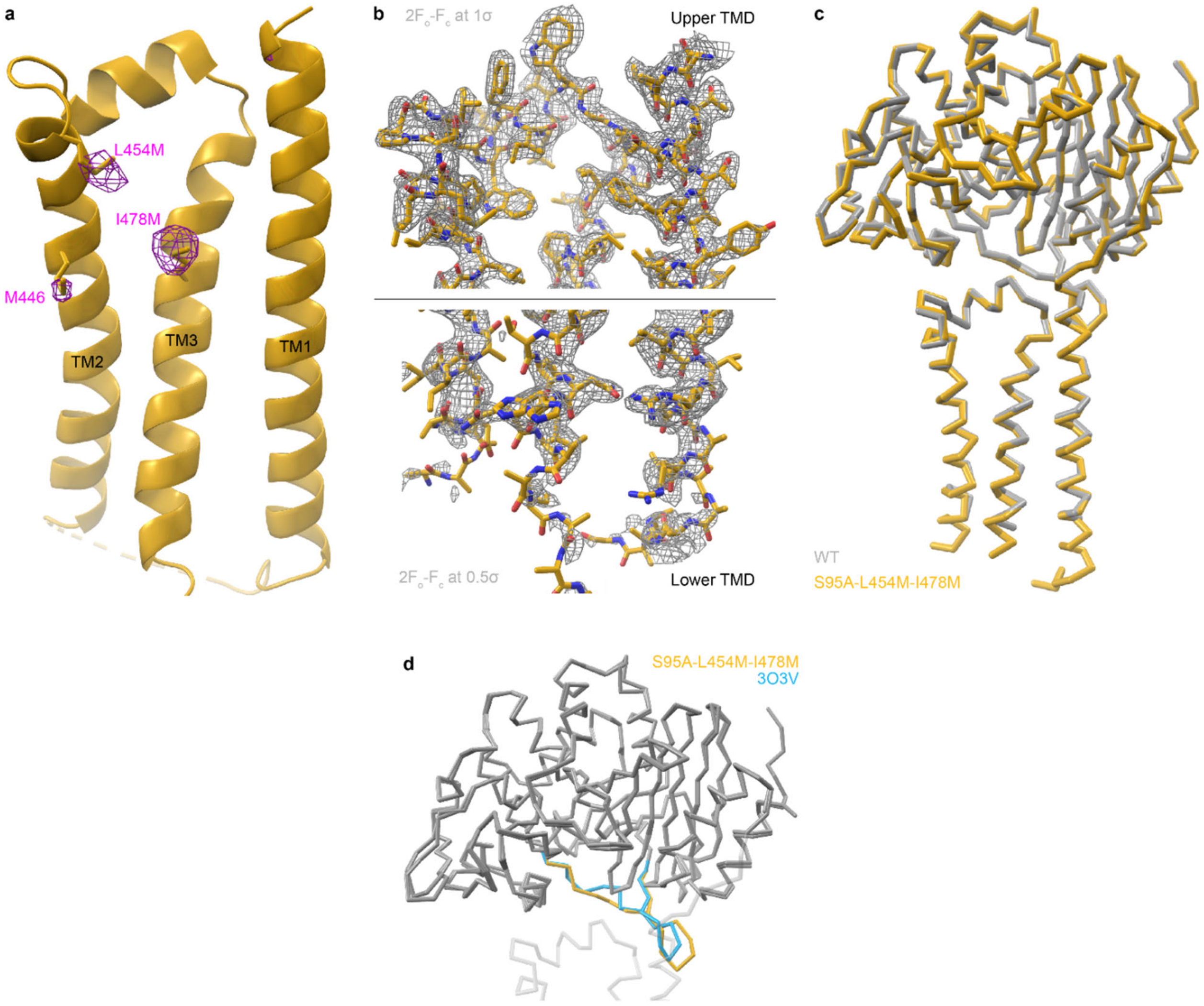

Supplementary Figure 1. Structure of full-length ClbP

a, Peaks from an anomalous difference Fourier map calculated from SeMet-substituted crystals match the positions of the methionines in the TMD and confirm the registry of our model (map contoured at 3σ). **b**, Sample $2F_o$-F_c maps show that parts of the TMD more proximal to the periplasmic domain (upper TMD) are better resolved than parts closer to the cytoplasm (lower TMD). **c**, Superposition of the structures for product-bound S95A-L454M-I478M (yellow) and wildtype ClbP (gray; companion paper[1]) showing that the two structures are nearly identical and that the introduced methionines do not affect the overall fold of the protein (RMSD = 0.37 over 436 shared Cα atoms). **d**, The periplasmic domain in our full-length structure is virtually identical to the structure of the isolated periplasmic domain published previously (PDB ID: 3O3V; RMSD 0.33 Å over 288 Cα atoms). The superposition of the two structures highlights differences in position of the β3-β4 loop (colored yellow in the product-bound S95A-L454M-I478M structure and cyan in the isolated periplasmic domain structure).

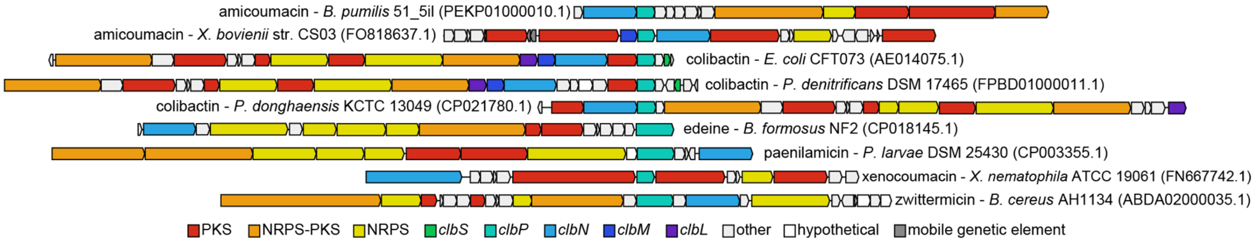

Supplementary Figure 2. Sample biosynthetic gene clusters

Biosynthetic gene clusters (BGCs) for natural products that utilize ClbN/ClbP systems for prodrug biosynthesis and prodrug removal, including amicoumacin[2,3], colibactin[4-6], paenilamicin[7], xenocoumacin[8], and zwittermicin[9,10]. While not previously identified as such, analysis of the edeine biosynthetic gene cluster[11-13] indicates that EdeA is likely also a true prodrug peptidase; we predict that EdeP is responsible for the biosynthesis of a previously unidentified acyl-D-Asn prodrug moiety (based on a condensation domain with homology to equivalent modules from surfactin, lichenysin, and colibactin; an adenylation domain predicted to have specificity for Asn; and an epimerase domain, required to produce D-Asn), as opposed to acting on 2,6-diamino-7-hydroxyazaleic acid as previously proposed[12]. In support of this, work in an accompanying paper uses a ClbP inhibitor to enrich several putative preedeines with colibactin-like prodrug moieties[1]. Additionally, a previously unidentified set of *clb*-like gene clusters are found in a subset of *Paenibacillus* species. These exhibit more genomic rearrangement than the *clb* clusters found in *Pseudovibrio* strains[61], and lack genes encoding ClbM and ClbS homologs, although a ClbL-like amidase is present, suggesting that formation of a colibactin pseudodimer is still plausible.

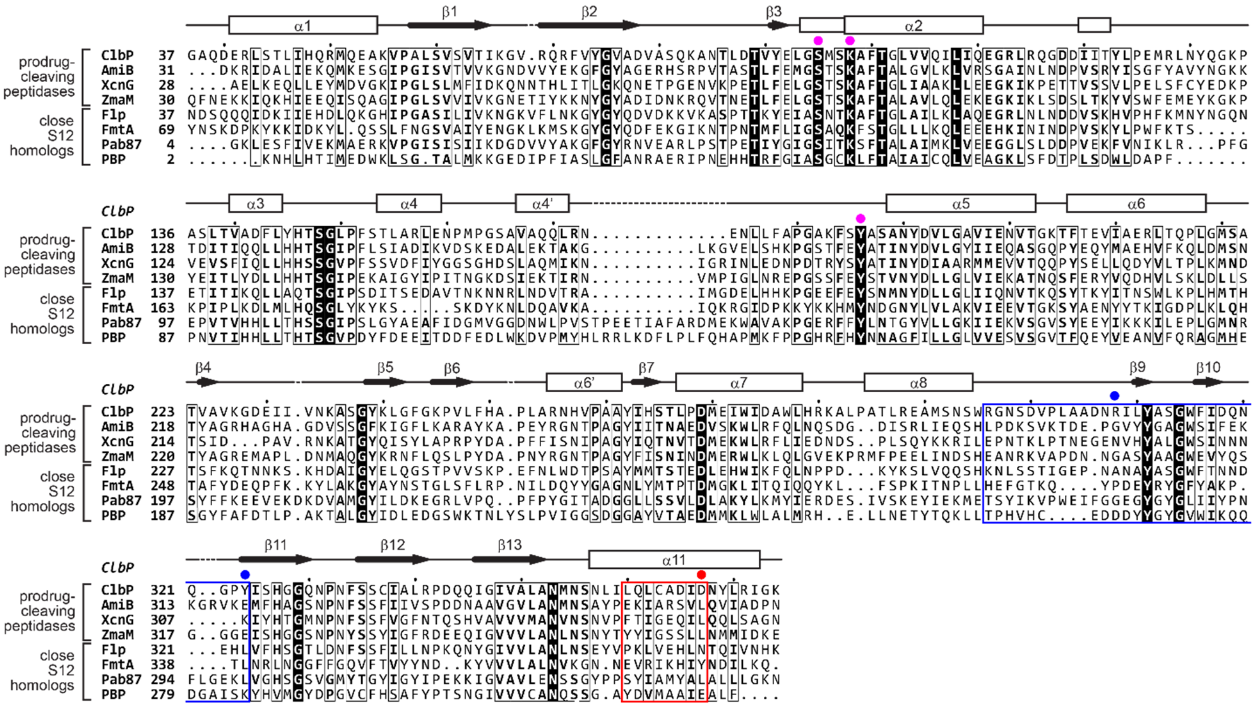

Supplementary Figure 3. Alignment of representative homologs.

Alignment of representative prodrug-activating peptidase (top) and close S12 homologs with structures available (bottom; see Extended Data Fig. 9b). The blue and red boxes denote the secondary structures involved in dimerization detailed in Extended Data Fig. 9. Magenta dots mark the catalytic triad residues, red and blue dots the interface residues targeted for mutagenesis. The alignment shows a lack the primary sequence conservation of the ClbP dimerization interface among other prodrug-activating peptidases as well as among closely related S12 homologs with structures deposited in the PDB.

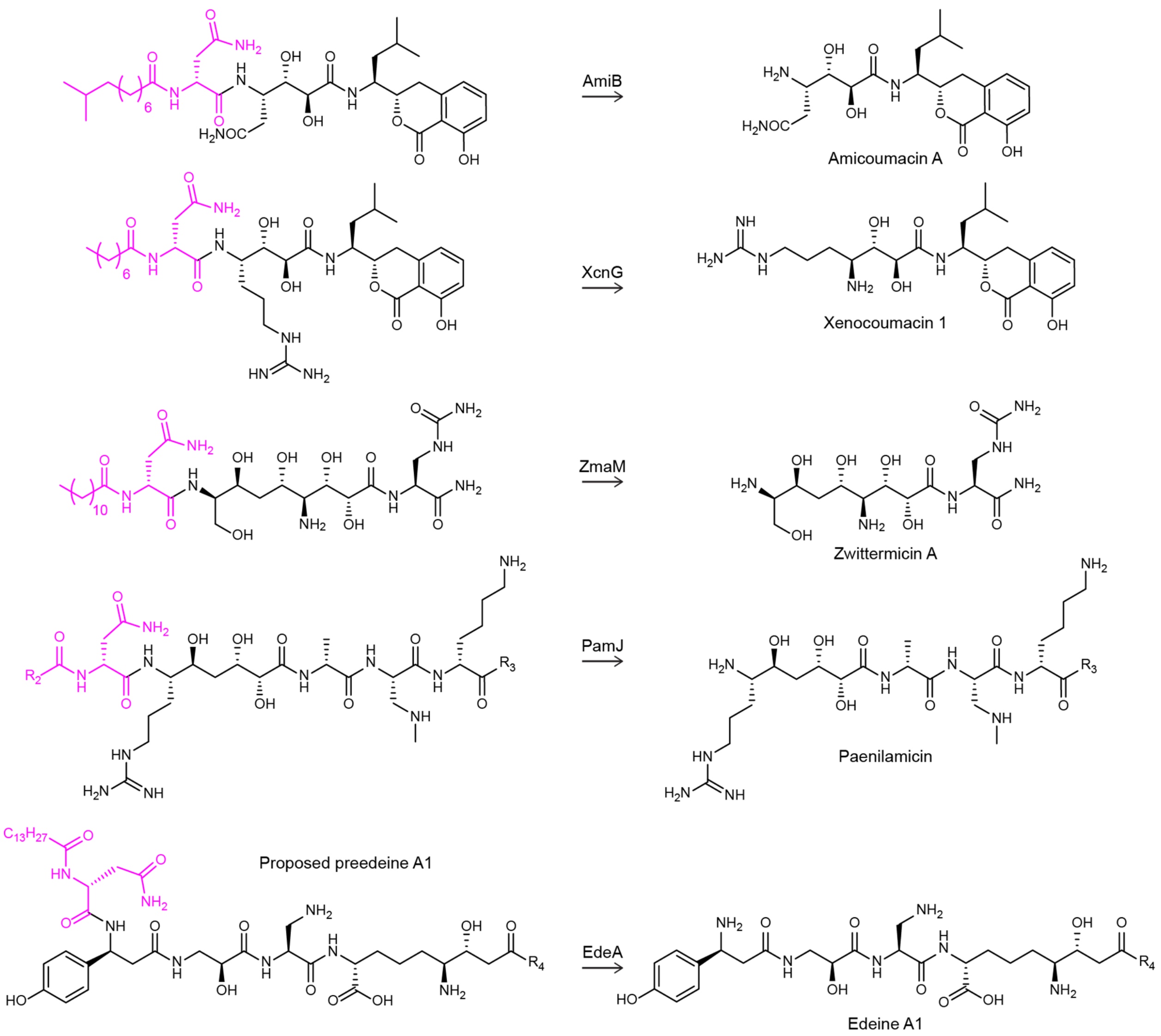

Supplementary Figure 4. ClbP is the only prodrug-activating peptidase proposed to process a pseudodimeric substrate

Chemical structure diagrams detailing the chemical reactions catalyzed by the amicoumacin[14], xenocoumacin[15], zwittermicin[9], and paenilamicin[16] peptidases—AmiB, XcnG, ZmaM, and PamJ respectively. Unlike ClbP, which is proposed to cleave a dimeric substrate (see Figure 1a), these peptidases are proposed to process monomeric precursors of their respective toxins. In all diagrams the *N*-acyl-D-asparagine prodrug motif cleaved from the precursor is colored in magenta. The R1 group in prezwittermicin represents a lauryl chain, as recently observed[1]. The R2 group in prepaenilamicin represents unknown acyl chains attached to their prodrug motifs, while the R3 in prepaenilamicin and paenilamicin represents a chemical structure derived from galantinic acid, *N*-methyl-diaminopropionic acid, glycine, and spermidine building blocks[16]. We further hypothesize that edeine biosynthesis employs a prodrug resistance mechanism and that EdeA is the activating peptidase. A proposed structure for a recently observed edeine A1 precursor containing a myristoyl-D-asparagine prodrug motif is shown[1], with R4 representing a glycylspermidine moiety[12].

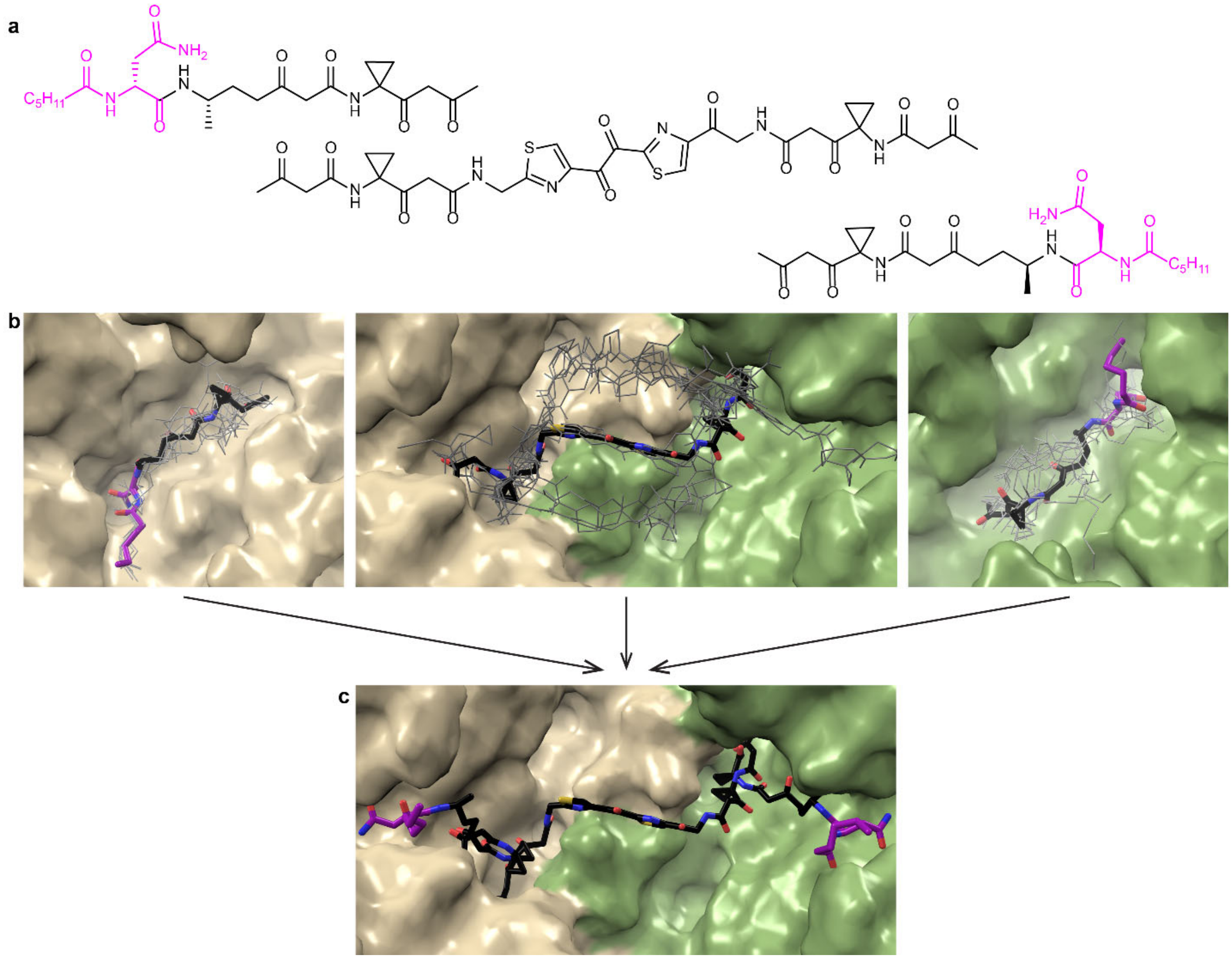

Supplementary Figure 5. Docking experiments to model precolibactin binding to ClbP

a, Chemical structure of the three overlapping fragments used to dock precolibactin onto the ClbP dimer. **b**, The panels show the selected pose (in sticks) for each of the three fragments along with alternative poses (gray lines). The selected pose for the two end fragments of precolibactin is shown with the nine other top scoring poses. Docking of the central fragment resulted in poses that either bound to a single subunit or that bridged both subunits. The selected pose for the central fragment is shown with other poses that bridge the two subunits and which reflect each of the three grooves that line the surface between the two active sites in dimeric ClbP. The selected pose for the central fragment was not among the top scoring poses, but it places the chemical groups that overlap with the two end fragments in proximity to their selected poses and therefore allowed us to connect the three fragments to generate a complete model of bound precolibactin. **c**, The selected pose for each of the three fragments are shown in the ClbP dimer cavity. Note that the docked molecules contain hexanoyl chains in place of the natural myristoyl chains.

Supplementary Note

Synthesis and validation of compound 1

NMR chemical shifts are reported in parts per million downfield from tetramethylsilane using the solvent resonance as internal standard for ^{1}H (DMSO-d$_6$ = 2.50 ppm) and ^{13}C (DMSO-d$_6$ = 39.52 ppm). Data are reported as follows: chemical shift, integration multiplicity (s = singlet, d = doublet, t = triplet, q = quartet, quint = quintet, m = multiplet), coupling constant, integration, and assignment. All solvents for synthesis were obtained from Sigma-Aldrich. All NMR solvents were purchased from Cambridge Isotope Laboratories (Tewksbury, MA). NMR spectra were collected in the Larkin-Purcell Instrumentation Center in Harvard University Department of Chemistry and Chemical Biology and visualized and processed using MestreNova, version 14.1.1-24571 (Mestrelab Research S.L., Escondido, CA). Preparative HPLC purification was run on a Dionex Ultimate 3000 instrument (Thermo Scientific) using Hypersil GOLDaQ column (250 mm x 20 mm, 5 μm particle size, Thermo Scientific).

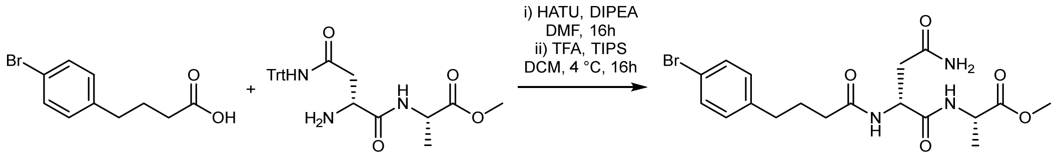

Compound **1**: In an oven-dried round-bottom flask, methyl *N*4-trityl-d-asparaginyl-l-alaninate (94 mg, 0.2 mmol, 1 equiv), 4-(4-bromophenyl)butanoic acid (59.7 mg, 0.24 mmol, 1.2 equiv), and 1-[Bis(dimethylamino)methylene]-1H-1,2,3-triazolo[4,5-b]pyridinium 3-oxide hexafluorophosphate (HATU, 93 mg, 0.24 mmol, 1.2 equiv) were dissolved in anhydrous dimethylformamide (DMF, 1.02 mL). Diisopropylethylamine (DIPEA, 89 mL, 0.51 mmol, 2.5 equiv) was added and the mixture stirred at room temperature overnight under a nitrogen atmosphere. The reaction mixture was then diluted with 10 mL 0.1N HCl and extracted with 10

mL ethyl acetate three times. The combined organic layers were washed with 5% lithium chloride, saturated sodium bicarbonate and saturated sodium chloride. The organic layer was then dried over anhydrous sodium sulfate and concentrated *in vacuo* to afford 132 mg white solid (94% crude yield). The crude intermediate was then immediately redissolved in DCM (3.8 mL) and cooled to 4°C. Triisopropylsilane (395 mL, 1.93 mmol, 10 equiv.) and trifluoroacetic acid (1.8 mL, 23 mmol, 120 equiv.) were added and the mixture stirred at 4°C overnight. The mixture was then concentrated *in vacuo* and the residue dissolved in DMSO and purified by preparative HPLC. The compound was eluted using the following gradient: 50% Solvent A for 2.5 minutes, gradient to 95% Solvent A over 7.5 minutes, hold at 95% Solvent A for 11.5 minutes, gradient to 50% solvent A over 1 minute, hold at 50% solvent A for 2.5 minutes (solvent A: HPLC-grade acetonitrile (VWR, HiPerSolv-Chromanorm) + 0.1% formic acid; solvent B: water + 0.1% formic acid; flow rate: 8 mL/minute; injection volume: 200 to 400 μL). The appropriate fractions were pooled and concentrated *in vacuo* to afford a white solid (30 mg, 33% yield over 2 steps). ^{1}H-NMR (500 MHz, DMSO-*d6*): δ (ppm) = 8.10 (d, J = 7.3 Hz, 1H), 7.97 (d, J = 8.1 Hz, 1H), 7.49 – 7.42 (m, 2H), 7.26 (s, 1H), 7.19 – 7.12 (m, 2H), 6.86 (s, 1H), 4.60 (td, J = 8.1, 5.7 Hz, 1H), 4.25 (quint, J = 7.2 Hz, 1H), 3.60 (s, 3H), 2.54 (d, J = 7.6 Hz, 2H), 2.33 (dd, J = 15.3, 8.2 Hz, 1H)†, 2.11 (t, J = 7.4 Hz, 2H), 1.76 (quint, J = 7.5 Hz, 2H), 1.25 (d, J = 7.3 Hz, 3H). ^{13}C-NMR (126 MHz, DMSO-*d6*): δ (ppm) = δ 173.3, 172.2, 171.7, 171.5, 141.8, 131.5, 131.2, 119.1, 52.3, 49.8, 48.0, 37.8, 34.9, 34.2, 27.2, 17.6. HRMS (ESI): calculated for $C_{18}H_{25}BrN_3O_5$ $[M+H]^+$, 442.0972; found 442.0969.

†A second peak matching this splitting pattern and integration (dd, J = 15.3, 8.2 Hz, 1H) appears at δ = 2.50 ppm in the ^{1}H-NMR, overlapping with the DMSO solvent residual peak. These peaks correspond to the two hydrogen atoms attached to the methylene carbon on the asparagine

sidechain. A ^{1}H-^{13}C HSQC was used to confirm the presence of the peak at 2.50 ppm and that these two peaks share an identical carbon coupling (see ^{1}H-^{13}C HSQC spectrum and inset below).

NMR spectra

Compound **1**: ^{1}H NMR (500 MHz DMSO-d_6)

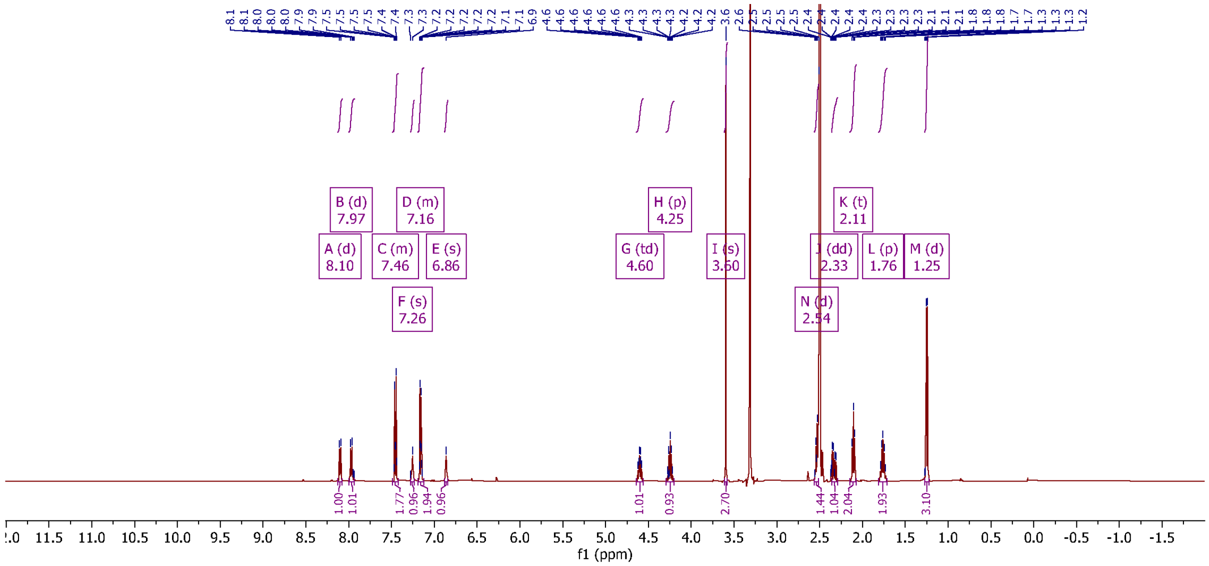

^{13}C NMR (126 MHz, DMSO-d_6)

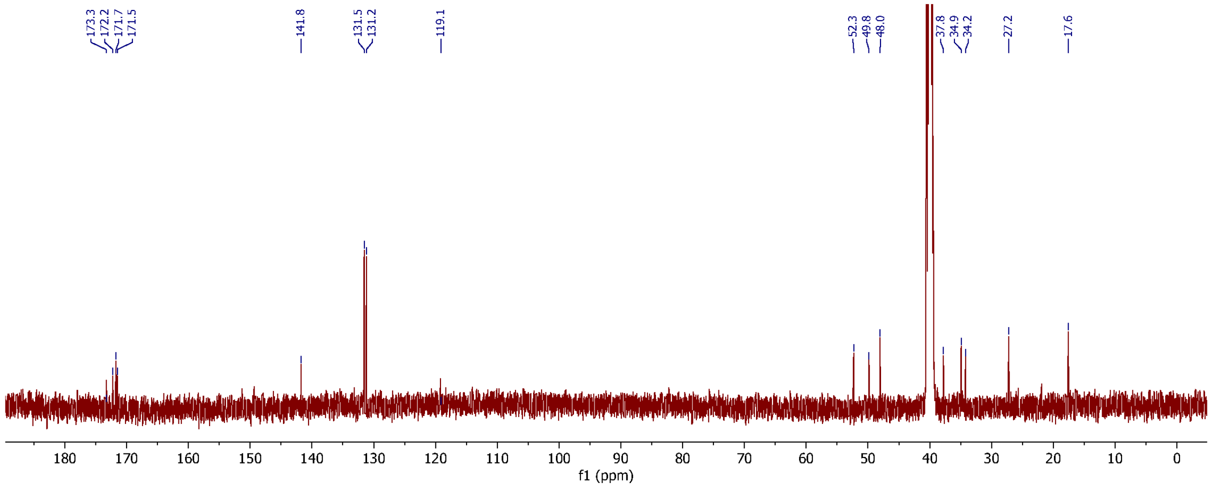

^{1}H-^{13}C HSQC (500 MHz, DMSO-d_6)

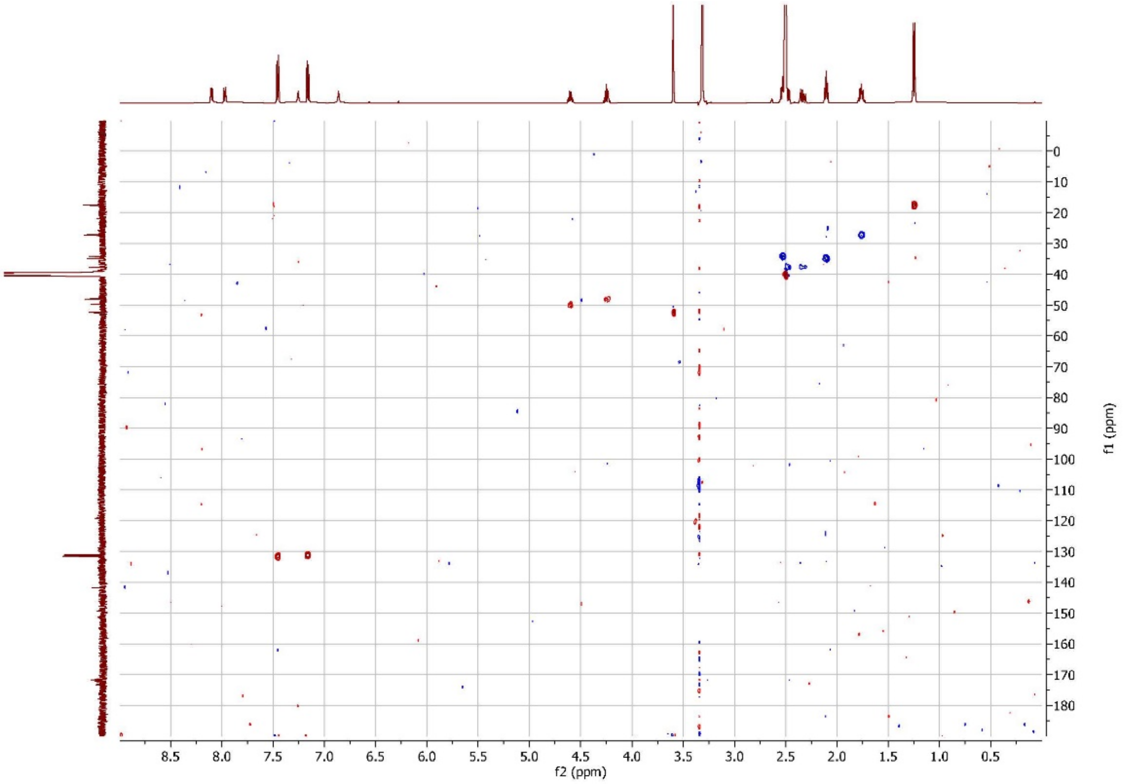

Inset focusing on the peaks corresponding to H-7 and H-19

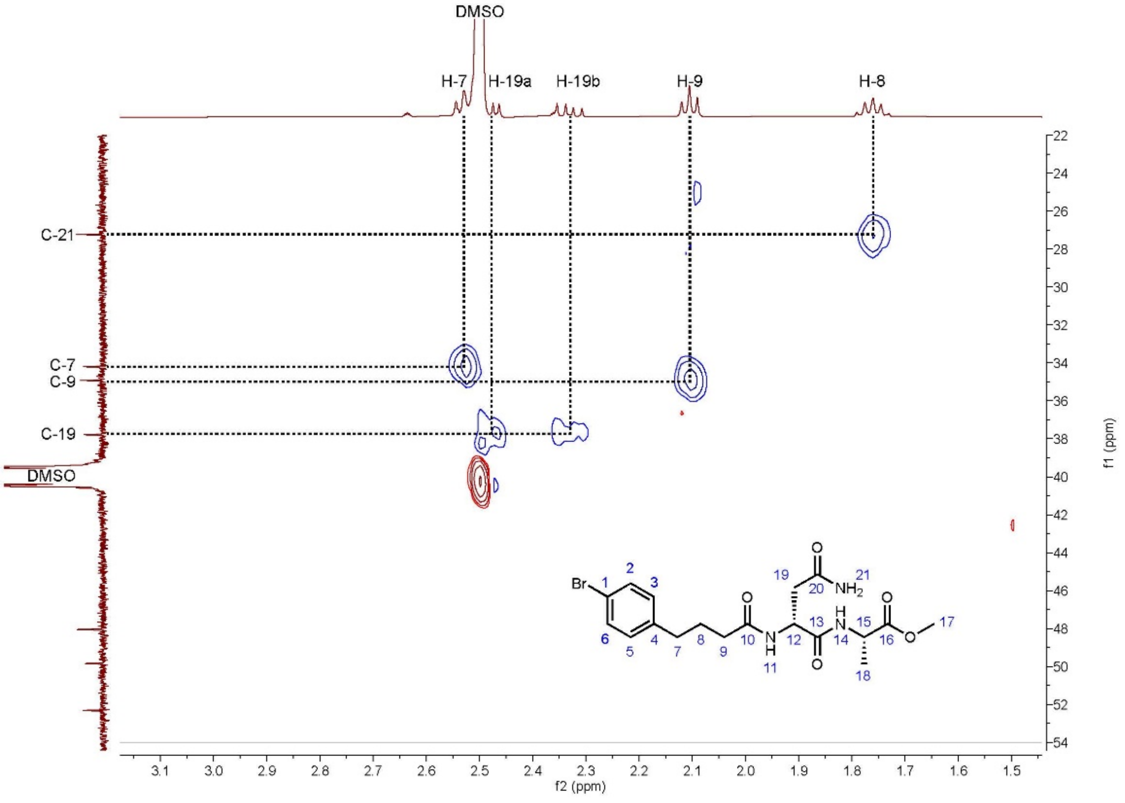

Supplementary References

1. Volpe, M.R. et al. A small molecule inhibitor prevents gut bacterial genotoxin production. *Nat Chem Biol*, in press (2022).

2. Park, H.B., Perez, C.E., Perry, E.K. & Crawford, J.M. Activating and Attenuating the Amicoumacin Antibiotics. *Molecules* **21**(2016).

3. Terekhov, S.S. et al. Ultrahigh-throughput functional profiling of microbiota communities. *Proc Natl Acad Sci U S A* **115**, 9551-9556 (2018).

4. Nougayrede, J.P. et al. *Escherichia coli* induces DNA double-strand breaks in eukaryotic cells. *Science* **313**, 848-51 (2006).

5. Putze, J. et al. Genetic structure and distribution of the colibactin genomic island among members of the family *Enterobacteriaceae*. *Infect Immun* **77**, 4696-703 (2009).

6. Sarshar, M. et al. Genetic diversity, phylogroup distribution and virulence gene profile of *pks* positive *Escherichia coli* colonizing human intestinal polyps. *Microb Pathog* **112**, 274-278 (2017).

7. Garcia-Gonzalez, E. et al. Biological effects of paenilamicin, a secondary metabolite antibiotic produced by the honey bee pathogenic bacterium *Paenibacillus larvae*. *Microbiologyopen* **3**, 642-56 (2014).

8. Park, D. et al. Genetic analysis of xenocoumacin antibiotic production in the mutualistic bacterium *Xenorhabdus nematophila*. *Mol Microbiol* **73**, 938-49 (2009).

9. Kevany, B.M., Rasko, D.A. & Thomas, M.G. Characterization of the complete zwittermicin A biosynthesis gene cluster from *Bacillus cereus*. *Appl Environ Microbiol* **75**, 1144-55 (2009).

10. Luo, Y. et al. Validation of the intact zwittermicin A biosynthetic gene cluster and discovery of a complementary resistance mechanism in *Bacillus thuringiensis*. *Antimicrob Agents Chemother* **55**, 4161-9 (2011).

11. Chen, W. et al. Draft genome sequence of *Brevibacillus brevis* strain X23, a biocontrol agent against bacterial wilt. *J Bacteriol* **194**, 6634-5 (2012).

12. Westman, E.L., Yan, M., Waglechner, N., Koteva, K. & Wright, G.D. Self resistance to the atypical cationic antimicrobial peptide edeine of *Brevibacillus brevis* Vm4 by the N-acetyltransferase EdeQ. *Chem Biol* **20**, 983-90 (2013).

13. Johnson, E.T., Bowman, M.J. & Dunlap, C.A. *Brevibacillus fortis* NRS-1210 produces edeines that inhibit the in vitro growth of conidia and chlamydospores of the onion pathogen *Fusarium oxysporum* f. sp. *cepae*. *Antonie Van Leeuwenhoek* **113**, 973-987 (2020).

14. Li, Y. et al. Directed natural product biosynthesis gene cluster capture and expression in the model bacterium *Bacillus subtilis*. *Sci Rep* **5**, 9383 (2015).

15. Reimer, D., Pos, K.M., Thines, M., Grun, P. & Bode, H.B. A natural prodrug activation mechanism in nonribosomal peptide synthesis. *Nat Chem Biol* **7**, 888-90 (2011).

16. Muller, S. et al. Paenilamicin: structure and biosynthesis of a hybrid nonribosomal peptide/polyketide antibiotic from the bee pathogen *Paenibacillus larvae*. *Angew Chem Int Ed Engl* **53**, 10821-5 (2014).
