# Supplementary material for: Structural basis of colibactin activation by the ClbP peptidase
Source: Nat Chem Biol. 2022 Oct 17;19(2):151–8. doi: 10.1038/s41589-022-01142-z (PMC9889268; doi:10.1038/s41589-022-01142-z)
Supplement: Supplementary file 2 — Reporting Summary [file 41589_2022_1142_MOESM2_ESM.pdf]

nature research

Corresponding author(s): Rachelle Gaudet

Last updated by author(s): Apr 27, 2022

Reporting Summary

Nature Research wishes to improve the reproducibility of the work that we publish. This form provides structure for consistency and transparency in reporting. For further information on Nature Research policies, see our Editorial Policies and the Editorial Policy Checklist.

Statistics

For all statistical analyses, confirm that the following items are present in the figure legend, table legend, main text, or Methods section.

n/a	Confirmed	
☐	☒	The exact sample size (*n*) for each experimental group/condition, given as a discrete number and unit of measurement
☐	☒	A statement on whether measurements were taken from distinct samples or whether the same sample was measured repeatedly
☒	☐	The statistical test(s) used AND whether they are one- or two-sided<br>*Only common tests should be described solely by name; describe more complex techniques in the Methods section.*
☒	☐	A description of all covariates tested
☒	☐	A description of any assumptions or corrections, such as tests of normality and adjustment for multiple comparisons
☐	☒	A full description of the statistical parameters including central tendency (e.g. means) or other basic estimates (e.g. regression coefficient) AND variation (e.g. standard deviation) or associated estimates of uncertainty (e.g. confidence intervals)
☒	☐	For null hypothesis testing, the test statistic (e.g. F, t, r) with confidence intervals, effect sizes, degrees of freedom and P value noted<br>*Give P values as exact values whenever suitable.*
☒	☐	For Bayesian analysis, information on the choice of priors and Markov chain Monte Carlo settings
☒	☐	For hierarchical and complex designs, identification of the appropriate level for tests and full reporting of outcomes
☒	☐	Estimates of effect sizes (e.g. Cohen's *d*, Pearson's *r*), indicating how they were calculated

Our web collection on statistics for biologists contains articles on many of the points above.

Software and code

Policy information about availability of computer code

Data collection	X-ray data collection: NE-CAT Beamline 24-ID-C; CryoEM data collection: The Harvard Cryo-EM Center for Structural Biology Titan Krios G3i Microscope.
Data analysis	Crystallography: XDS 0.86, AIMLESS 7.0.077, PHENIX 1.19.1-4122, COOT 0.9.4; Docking: Cresset Flare version 3.0.0; NMR: MestreNova, version 14.1.1-24571; Sequence analyses: TMHMM 2.0, RASTtk 1.3.0, InterProScan 5.50-84.0, PKS/NRPS Analysis 1.1, antiSMASH 6.0, PRISM 4.4.5, Cytoscape 3.8.2, JackHMMER 3.3.2, Clustal Omega 1.2.4, WebLogo 3, CDHit 4.8.1, PhyML v3.1, Archaeopteryx v2.0.0a4 ; CryoEM: SerialEM 3.8.6, MotionCor2 1.2.6, CTFFIND4 4.1.13, crYOLO 1.7.5, cryoSPARC 3.2, RELION 3.0.4, COOT 0.9.3, ISOLDE 1.0b4.dev0, UCSF Chimera 1.15, PHENIX 1.20.1-4487.

For manuscripts utilizing custom algorithms or software that are central to the research but not yet described in published literature, software must be made available to editors and reviewers. We strongly encourage code deposition in a community repository (e.g. GitHub). See the Nature Research guidelines for submitting code & software for further information.

Data

Policy information about availability of data

All manuscripts must include a data availability statement. This statement should provide the following information, where applicable:

- Accession codes, unique identifiers, or web links for publicly available datasets
- A list of figures that have associated raw data
- A description of any restrictions on data availability

Atomic coordinates and structure factors for the reported crystal structures in this work have been deposited to the Protein Data Bank under accession numbers 7MDE (Monoolein-bound S95A-L454M-I478M (SeMet) ClbP) and 7MDF (Product-bound S95A-L454M-I478M ClbP). Corresponding X-ray diffraction images have been deposited to the SBGrid Data Bank under accession numbers 833 (doi:10.15785/SBGRID/833) and 831 (doi:10.15785/SBGRID/831), respectively. The map of

the cryo-EM reconstruction has been deposited to the Electron Microscopy Data Bank (EMDB) (accession number: EMD-26593), and the refined coordinates to the Protein Data Bank (PDB ID: 7UL6). The sequences for bioinformatic analyses were procured from PFAM (seed alignment version 33.1), UniProt (2021_02 release), GenBank (release 242), ENA (2021.03.03) and MEROPS (12.4), and the dataset (SSN, aligned sequences, and phylogenetic tree) is in Supplementary Data. Source data for Figures 2 and 3, and Extended Data Figures 3, 4, 6, and 8 are provided with this paper.

Field-specific reporting

Please select the one below that is the best fit for your research. If you are not sure, read the appropriate sections before making your selection.

☒ Life sciences ☐ Behavioural & social sciences ☐ Ecological, evolutionary & environmental sciences

For a reference copy of the document with all sections, see nature.com/documents/nr-reporting-summary-flat.pdf

Life sciences study design

All studies must disclose on these points even when the disclosure is negative.

Sample size	The number of crystals used to determine each structure (as indicated in Table 1) were chosen to insure >99% overall completeness of the x-ray diffraction datasets.
Data exclusions	No data were excluded.
Replication	All assays were performed at least in triplicate as noted. All attempts at replication were successful.
Randomization	R-free flags were chosen at random using the default function to do so in the PHENIX Reflection file editor for the first structure (7MDE). The flags were transferred (and extended as needed, at random using the PHENIX Reflection file editor) to the other structure to minimize model bias. For the other experiments randomization is not relevant: we were not collecting data with samples from different sources that would require randomization to avoid biases in the data, and therefore no randomization was implemented.
Blinding	The R-free set was selected at random by the PHENIX refinement software, with the investigators blind to the selection. For the other experiments blinding is not relevant and was not used because unintentional bias cannot affect the results for the type of data that we collected.

Reporting for specific materials, systems and methods

We require information from authors about some types of materials, experimental systems and methods used in many studies. Here, indicate whether each material, system or method listed is relevant to your study. If you are not sure if a list item applies to your research, read the appropriate section before selecting a response.

Materials & experimental systems

n/a	Involved in the study	
☒	☐	Antibodies
☒	☐	Eukaryotic cell lines
☒	☐	Palaeontology and archaeology
☒	☐	Animals and other organisms
☒	☐	Human research participants
☒	☐	Clinical data
☒	☐	Dual use research of concern

Methods

n/a	Involved in the study	
☒	☐	ChIP-seq
☒	☐	Flow cytometry
☒	☐	MRI-based neuroimaging
